# Influence of phosphate fertilizers on the radioactivity of agricultural soils and tobacco plants in Kenya, Tanzania, and Uganda

**DOI:** 10.1007/s11356-023-27543-8

**Published:** 2023-06-20

**Authors:** Dennis A. Mwalongo, Nils H. Haneklaus, Fernando P. Carvalho, Jacob B. Lisuma, Thomas T. Kivevele, Kelvin M. Mtei

**Affiliations:** 1 Tanzania Atomic Energy Commission, Directorate of Nuclear Technology and Technical Services, P.O. Box 743, Arusha, Tanzania; 2 Technische Universität Bergakademie Freiberg, Leipziger Straße 29, 09599 Freiberg, Germany; 3 Nelson Mandela African Institution of Science and Technology (NM-AIST), School for Materials, Energy, Water, Environmental Science and Engineering, P.O. Box 447, Arusha, Tanzania; 4 Universität für Weiterbildung Krems, Td-Lab Sustainable Mineral Resources, Dr.-Karl-Dorrek-Straße 30, 3500 Krems an der Donau, Austria; 5 Laboratório de Protecção e Segurança Radiológica, Instituto Superior Técnico/Campus Tecnológico Nuclear, Universidade de Lisboa, Lisboa, Portugal; 6 Tobacco Research Institute of Tanzania (TORITA), P.O. Box 431, Tabora, Tanzania

**Keywords:** Radionuclides, Gamma-ray spectrometry, NPK fertilizer, Tobacco, Radiation, Hazard indices, Excess cancer risk

## Abstract

Three brands of NPK fertilizers that contain variable concentrations of natural radioactivity are commonly used in tobacco plantations in Kenya, Tanzania, and Uganda. Tobacco plants are known for hyper-accumulation of natural radionuclides, particularly ^238^U. This study investigated if the elevated radioactivity in phosphate fertilizers could enhance radioactivity in soils and tobacco plant leaves. The ^232^Th, ^238^U, and ^40^K radionuclide levels in NPK-fertilized soils and tobacco leaves were measured using gamma-ray spectroscopy. The research included a one-year reference experiment with tobacco growing in plots, a ten-year semi-controlled experiment in well-managed tobacco farms, and a field survey of radioactivity in soils and tobacco leaves at three traditional tobacco fields in Migori (Kenya), Urambo (Tanzania), and Kanungu (Uganda). The findings demonstrated that soils and tobacco leaves exposed to NPK fertilizers with increased radioactivity had activity concentrations of ^232^Th, ^238^U, and ^40^K that were considerably higher (at all sites) than in the control samples (with no use of NPK fertilizers). As the continued application of NPK fertilizers raises concentrations of ^232^Th, ^238^U, and ^40^K in agricultural soils, the study assessed radiological risks for humans from exposure to agricultural soils enriched with phosphate fertilizers, and it was found to be below the exposure limit of 1 mSvy^-1^ suggested by the International Commission on Radiological Protection (ICRP). However, tobacco consumers, both by snuffing and smoking, may face significant radiological risks, as the snuffing and smoking resulted in effective doses that were 2.41 to 6.53 and 1.14 to 2.45 times greater than the average yearly dose that the general public receives from inhalation of natural radionuclides (United Nations Scientific Committee on Atomic Radiations estimates). Furthermore, the results indicate that the lifetime excess cancer risk for tobacco snuffers and smokers ranged from 5 × 10^-5^ to 24.48 × 10^-3^ and 2.0 × 10^-5^ to 9.18 × 10^-3^, respectively. The influence of phosphorus-derived fertilizer containing relatively high natural radioactivity, potential human radiation exposure, and radiological risk due to gamma radionuclides is estimated and discussed. The results reveal that applying phosphate fertilizers enhances natural radioactivity in soil and is subsequently influenced by soil to tobacco plant uptake. Therefore, the study recommends that countries use fertilizers with lower radionuclide content to conserve soil quality and reduce gamma-emitting radionuclides in tobacco plants.

## Introduction

The earth’s crust contains various concentrations of the radioactive elements ^238^U, ^232^Th, and ^40^K, which are all naturally occurring (Carvalho and Oliveira 2006; Gupta and Walther 2019). Phosphate ore, the raw material used to manufacture phosphate fertilizers, can contain naturally occurring radioactive elements such as uranium (^238^U, T_1/2_ = 4.47 × 10^9^ a), thorium (^232^Th, T_1/2_ = 14.05 × 10^9^ a), and potassium (^40^K, T_1/2_ = 1.25 × 10^9^ a) in increased concentrations depending on the origin and type of rock and fertilizer manufacturing procedure, but often in concentrations that can be considered elevated (IAEA 2013, 2018; Haneklaus 2021; Van Dung et al. 2022). The application of phosphate fertilizers can thus be a major source of radionuclides and heavy metals added to agricultural soils (Haneklaus et al. 2017; De Souza Braz et al. 2021).

The amounts of naturally occurring radioactive materials (NORMs) added in a single fertilizer application may be considered insignificant compared to the volume of receiving soil and its naturally occurring radioactive elements. Nonetheless, it has been noted that the prolonged and intense use of phosphate fertilizers in tobacco crops led to a considerable buildup of ^238^U, ^232^Th, and ^40^K in the soil (Khater 2012; Chauhan and Chauhan 2014; Bigalke et al. 2017, 2020; De Souza Braz et al. 2021). Moreover, following fertilizer application, the additional radionuclides ^238^U, ^232^Th, and ^40^K may move from the soil to other environmental compartments and represent a risk to human health through chemical and radiological exposure (Abed et al. 2022).

Natural radionuclides in fertilizers and agricultural soils have attracted the attention of researchers because of the potential radiation exposure to humans. For instance, Yamaguchi et al. (2009) examined the distribution of uranium in agricultural soil components following the long-term application of NPK fertilizer on rice paddies in Japan. They found that the uranium concentration was higher in NPK fertilized crops compared to the control sample. Takeda et al. (2006) reported that the uranium added with fertilizer application was concentrated in the top 35 cm of the soil stratum, with the soil organic compounds influencing uranium accumulation. In addition, the radioactive elements ^238^U, ^232^Th, and ^40^K are long-lived and may remain in the agricultural soil, contaminate groundwater, and can even be taken up by plants (Papastefanou 2009; Gupta et al. 2020).

Uranium uptake by plants depends on the plant species, the amount of uranium in soils, and climatic conditions (Asaduzzaman et al. 2015; Tagami and Uchida 2020). Several studies have indicated that ^238^U, ^232^Th, and ^40^K in the soil are not available for root uptake by most plant species. However, the tobacco plant (*Nicotiana tabacum* L.) has shown a distinct character as a hyper-accumulator of radionuclides from the soil compared to other plants, such as cereals and vegetables (Papastefanou 2009; Stojanovic et al. 2012; Kadhim and Ridha 2019; John et al. 2022).

Tobacco is a cash crop extensively grown in Kenya, Tanzania, and Uganda. Tanzania is a leading tobacco producer in the region, 3rd in Africa and 8th in the world (Ndomba 2018). Uganda is 2nd in East Africa, followed by Kenya, Rwanda, and Burundi (James 2019). In these countries, tobacco plantations intensively use nitrogen (N), phosphorous (P), and potassium (K) fertilizers from different brands (Lisuma et al. 2022b). Furthermore, Kenya, Tanzania, and Uganda are rural countries and more than 80% of the population depends on agriculture by cultivating various crops, including tobacco, for their livelihood (Lokuruka 2020, 2021). The fact that large populations in these countries are directly involved in agricultural production means that a large fraction of the population could potentially be exposed to radioactivity beyond natural background levels through the intensive application of phosphate fertilizers. However, systematic studies on natural radionuclides associated with fertilizer use in tobacco production in Kenya, Tanzania, and Uganda have to the best of our knowledge, not been performed yet, although different studies have determined elevated concentrations of radionuclides, mainly ^238^U in regionally produced and used mineral fertilizers in those countries (Makweba and Holm 1993; Banzi et al. 2000; Mwalongo et al. 2022).

It is worth mentioning that ^210^Po and its precursor ^210^Pb are primary sources of radiation exposure due to smoking (Carvalho and Oliveira 2006; Naina et al. 2010; Eke and Ishfaq 2021). Specifically, this study investigated the concentrations of ^238^U, ^232^Th, and ^40^K in fertilizers, agricultural soils, and tobacco plant leaves in relationship with the application of NPK fertilizers. In addition, the study also used analytical data to assess the radiological risk and potential impact on the health of agriculture workers of tobacco plantations and consumers of this tobacco.

## Material and methods

### Experimental design and location of study areas

Three experiments were monitored, which included one-year experiments, ten years of guided experimental farms monitored by the Tobacco Research Institute of Tanzania (TORITA), and smallholder farmer’s practices’ farms. The one-year experiment was a completely randomized design with four treatments. One district in each Kenya, Tanzania, and Uganda was selected for smallholder farmer’s practice study.

The tobacco plant was chosen to assess soil-to-plant transfer of radionuclides based on its hyper-accumulation property of radionuclides, especially of uranium. Furthermore, tobacco is grown using NPK fertilizers rich in the macro-nutrients: nitrogen (N), phosphorous (P), and potassium (K) (NPK 10:18:24). The YaraMila Compound (YC) N_10_P_18_K_24_ fertilizer contains three component nutrients: N, P, and K, while the YaraMila Blended (YB) N_10_P_18_K_24_ fertilizer was blended mechanically to supply balanced nutrients of N, P, and K individually (Lisuma and Mbwambo 2022). In addition, the Golden Leaf Tobacco (GLT) N_10_P_18_K_24_ fertilizer was blended from Minjingu organic Hyper-Phosphate (MoHP) rock containing significant concentrations of naturally occurring uranium (Mwalongo et al. 2022). Therefore, these mineral fertilizers can contain elevated concentrations of naturally occurring radionuclides associated with the phosphate ore processed for its P content.

Three studies were carried out. (1) A one year-control experiment with tobacco grown in plots was performed at the TORITA fields in western Tanzania in the 2021/2022 cropping season. (2) A long-term experiment was carried out in different tobacco fields in Tanzania under TORITA management for the previous ten years (from 2012/2013 to 2021/2022) and with annual tobacco cropping. (3) An assessment of radioactivity levels in traditional tobacco farms in the East Africa region was carried out in selected farms: the Urambo farm (Tanzania), Migori (Kenya), and Kanungu (Uganda).

#### The one year-controlled experiment

The experimental plots for the one-year controlled experiment were set up at the TORITA experimental farm in Tumbi (Tabora region) during the 2021/2022 cropping season. The tobacco plant *Nicotiana tabacum L.* (variety K326) was planted, and different brands of NPK fertilizers (NPK 10:18:24) were applied. The fertilizers used in this controlled experiment were YC, YB, and GLT. Tobacco growers used these NPK fertilizers during the experimental year. The first two fertilizers were imported from Sweden, while the last one was locally manufactured at the Minjingu Mines and Fertilizers Limited Company in Tanzania. After applying NPK fertilizers, calcium ammonium nitrate (CAN - 27%) was applied to the experimental plots after two weeks as it is usually done in tobacco plantations. This fertilizer is based on synthetized ammonia and is practically radioactivity free. The three phosphate fertilizers (YC, YB, and GLT) were analyzed for gamma-emitting radionuclides at the gamma-ray spectrometry laboratory at the department of technical support and radiation protection service of the Tanzania Atomic Energy Commission (TAEC) Northern zone in Arusha.

The experimental plots were set up in agricultural soil that had not been cultivated for about ten years (a forest before) and received no influence from phosphate fertilization. The site was prepared by clearing the forest, and the soil was prepared using hand hoes. Tobacco seeds (variety K326) were sown in four seedbeds with four treatments (without fertilizer, fertilized with YC, YB, and GLT), each with 1 m × 1 m size after application of 1 kg of respective NPK fertilizer per treatment as is the standard procedure (Lisuma et al. 2022a).

Seedling seedbeds and experimental plots were prepared for one cropping season. Each experimental plot was replicated three times, thus obtaining a total of 12 plots (three plots per treatment). A control plot (no fertilizer) was also set up. The ridge and fallow field plots were 6 m × 6 m in size, with a 2 m spacing between blocks and a 1 m spacing within plots. A 3-meter-wide pathway was established around the entire experimental area to reduce potential outside influences. After 10–14 days, germinated tobacco seedlings were transplanted to new seedbeds and then re-transplanted into the experimental plots 60 days after sowing with an intra-row spacing of 0.5 m × 0.5 m.

**Fig. 1:**
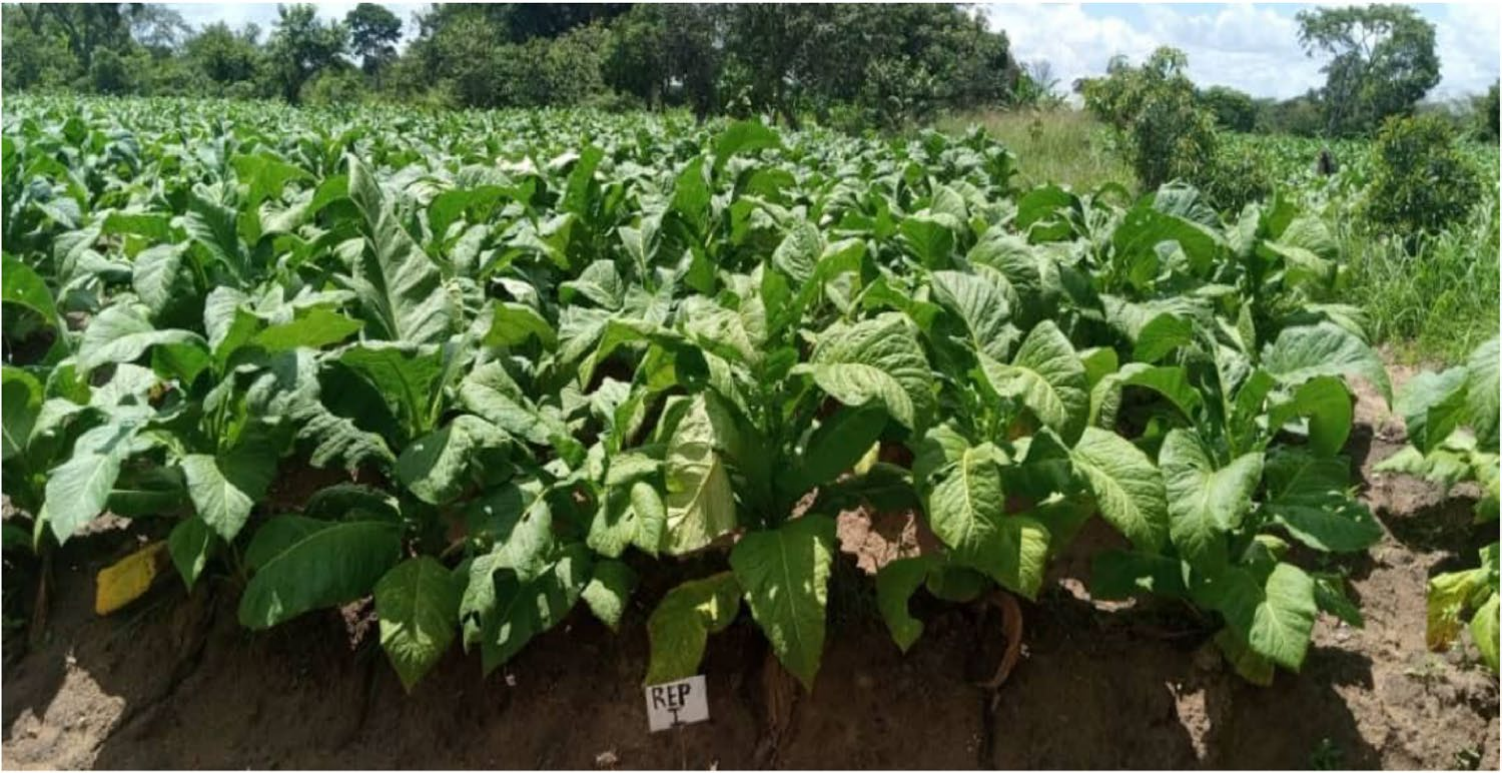
A tobacco experimental plot showing replication of the controlled experiment

Seven days after transplanting, the seedling’s growth was boosted with application of the three fertilizer brands YC, YB, and GLT in the respective plots, each with three replications, as shown in Fig. 1. Twenty-one days after seedling transplanting, the CAN fertilizer (27% N) was applied at the rate of 30 grams per plant and agronomic practices were managed throughout the experiment. The tobacco leaves were sampled 12–15 weeks after topping.

The lower, middle, and upper-end leaves were sampled and mixed to get a composite sample for each sampling point for radionuclide measurement. In addition, a composite soil sample, combining soil from the ridge and fallow, was taken from each plot after reaping the leaves. The leaves and soil samples were packed in labeled plastic bags and transported to the laboratory for further preparation and analysis.

#### Ten-year semi-controlled experiment

**Fig. 2:**
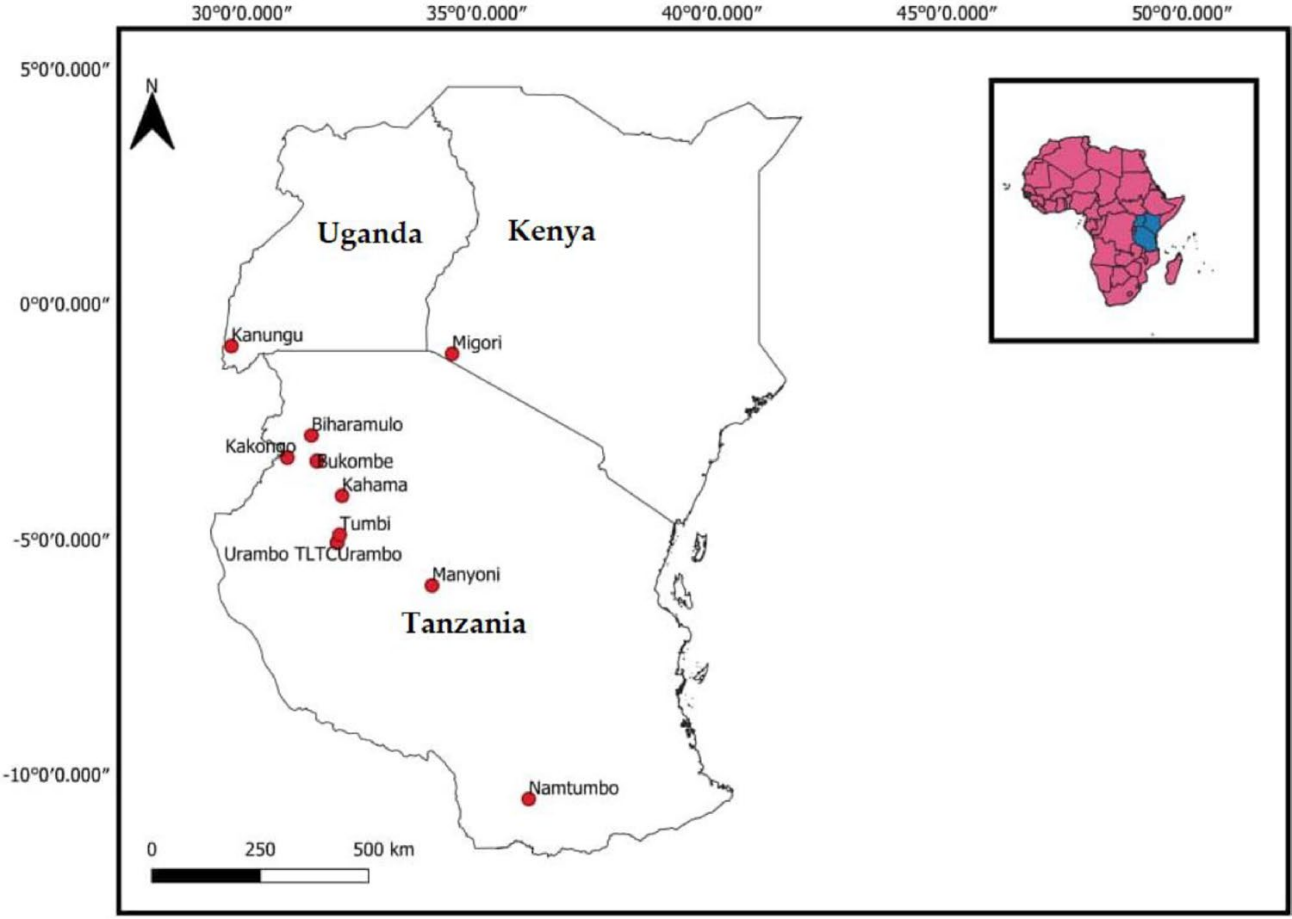
Map of Kenya, Tanzania, and Uganda showing the soil and tobacco leaf sampling sites

The experimental sites used in this investigation were tobacco farms managed under the supervision of TORITA for the previous ten years (ten cropping seasons). One of the core functions of TORITA is to perform long term monitoring of the practices and performances of tobacco plantations in Tanzania. The tobacco farms selected had applied over the previous decade NPK (10:18:24) fertilizers, including NPK derived from YB and GLT, during the year 2021/2022. Therefore, farming practices for ten years duration were slightly different to the one year-control experiments. The tobacco farms investigated were located in 7 districts in Tanzania: Urambo (Tabora), Kahama (Shinyanga), Bukombe (Geita), Biharamulo (Kagera), Kakonko (Kigoma), Manyoni (Singida), and Namtumbo (Ruvuma) as shown in Fig. 2.

#### Investigation in selected tobacco farms in Kenya, Tanzania, and Uganda

The experiments in the three countries were carried out to assess the radioactivity levels in long-standing tobacco farms representative of the traditional tobacco growing districts/counties in Kenya (Migori), Tanzania (Urambo farm), and Uganda (Kanungu). Farmers’ cultivation practices and NPK fertilizer application could not be controlled and might be slightly different from those followed in the one-year and ten-year experiments. These experimental sites at the three major tobacco producing sites were aimed at giving an actual picture of the radioactivity and radiation exposure in commercial tobacco farming. The districts/counties were chosen to represent leading tobacco producers. For instance, Urambo (Tanzania) accounts for over 50% of Tanzania’s tobacco production (Lisuma et al. 2022b). Migori produces over 80% of tobacco in Kenya (James 2019), and in Uganda, the Kanungu district is responsible for about 20% of the countries tobacco production (Wanyonyi et al. 2020; Chune et al. 2022). Soil and tobacco leaf samples were collected from these farms and analyzed by gamma spectrometry at the TAEC in Arusha.

### Sampling and sample preparation

Soil samples from a depth of 0–30 cm were collected using an open-end stainless-steel hand-held Auger corer (70 mm diameter) manufactured by Thermo-Fisher Scientific, Germany. Ten soil core samples were collected between the plants at 0–30 cm depth from each sampling site to make a layer of composite samples of about 500 g each. Larger plant debris and stones were removed, and soil samples were subsequently homogenized. Ten (10) tobacco leaves from the lower, middle, and upper portions were collected during the harvesting of the 2021/2022 cropping season. In the laboratory, the tobacco leaves were thoroughly washed with deionized water. Soil and tobacco samples were oven-dried at 80 °C until a constant weight was reached. The dried soil and tobacco leaves were ground and sieved using a 200-micron mesh screen, transferred to metal canisters, and sealed airtight. The average weight of the sample materials in the canisters for gamma spectrometry was approximately 250 g for soil samples and 186 g for tobacco leaf samples. The samples were stored for 30 days in the laboratory before measurements.

### Radionuclides analysis by gamma spectrometry

After 30 days of storage to allow for the formation of secular radioactive equilibrium of ^238^U and ^232^Th with their respective short-lived progenies, while ^40^K was measured from the 1640 keV distinct peak, the samples were counted for 36,000 s on a large volume high purity germanium (HPGe) gamma spectrometer (manufactured by ORTEC), inside lead shielding and connected to a multichannel analyzer. The Gamma Vision^®^ software was used in spectrum analysis (ORTEC 2020). The energy and efficiency calibration of the TAEC gamma spectrometry system was performed for lower and higher energies using multi-nuclide calibration sources type CBSS 2, Certificate No. 1035-Se-40202-17, Serial No. 270217-1621040, traceable to the Czech Metrology Institute. The average concentrations of radionuclides ^212^Pb (238.6 keV) and ^228^Ac (911.1 keV) were used to calculate the concentration of ^232^Th in the sample, while the peak gamma energies of radionuclides ^214^Bi (764.8 keV) were used to calculate the concentration of ^238^U. The gamma lines 609.3 keV and 1120 keV for the ^238^U series, and 583.2 keV, 727.3 keV, and 795 keV for the ^232^Th series were removed from the estimation of radionuclide activity concentration because they are impacted by coincidence summation (Newman et al. 2008; Polouckova 2021). The activity concentration for each radionuclide of interest was calculated using Eq. 1 (Kovacs et al. 2017):

Eq. 1 $$A=\frac{N}{\ {P}_{\upgamma}\times \upvarepsilon \times W}$$

where *A* is the activity per unit mass of dry weight of the sample in Bq kg^−1^, *N* is the net counts per second (cps), 𝑃_𝛾_ is a gamma-line emission probability of a particular radionuclide, ε is the gamma line emission intensity (%), and 𝑊 (kg) is the weight of the sample.

### Analytical quality control

Using the IAEA-375 Certified Reference Material (CRM) with soil matrix, the calibration of the spectrometry equipment was evaluated for accuracy and reliability. The outcomes are displayed in Table 1.

**Table 1:** Measured activity concentrations of IAEA-375 reference material and reference values

Radionuclides	Lab value (Bq kg^-1^)	IAEA value (Bq kg^-1^)	Relative bias (%)
^238^ U	18.4 ± 0.71	19.8 ± 0.6	-7%
^232^Th	469.2 ± 20.45	434.4 ± 18.9	8%
^40^K	427.6 ± 15.67	406.8 ± 16.7	5%

The relative bias between the IAEA certified reference values and measured results were calculated using Eq. 2

Eq. 2 $$\textrm{Relative}\ \textrm{bias}\ \left(\%\right)=\frac{\textrm{Laboratory}\ \textrm{Measured}\ \textrm{Value}-\textrm{IAEA}\ \textrm{reference}\ \textrm{Value}}{\textrm{IAEA}\ \textrm{reference}\ \textrm{Value}}\times 100$$

The results indicate that the overall bias was within ±8%, which indicate that the accuracy of the measurement method was suitable for the purpose of this research (Shakhashiro et al. 2008).

### Physical-chemical characteristics of soils

The physical-chemical characteristics of the soil samples, namely, cation exchange capacity (CEC), organic matter content (OM), soil pH, and soil textures (sand, silt, and clay), were determined to the depth of 0-30 cm using standard scientific methods described by Moberg (2001).

### Calculation of radiological parameters

It was hypothesized that radionuclides present in soil and tobacco leaves could be a radiological hazard to farm workers and members of the public. Furthermore, tobacco consumers may ingest and inhale the radionuclides contained in the tobacco leaves (IAEA 2013). Therefore, the radioanalytical results of the three primordial radionuclides were used to quantify several radiological hazard indexes that are briefly introduced in the following subchapters.

#### Radium equivalent activity in soil

Radium equivalent (Ra_eq_) is a radiological health risk parameter resulting from exposures to naturally occurring radioactive materials containing ^232^Th, ^238^U, and ^40^K. It is used by regulatory bodies to set a single regulatory limit rather than setting individual radionuclide limits (Abed et al. 2022). The radium equivalent (Ra_eq_) for soil samples was calculated using Eq. 3 (Tufail 2012):

Eq. 3 $${Ra}_{eq}={A}_U+1.43{A}_{Th}+0.077{A}_K$$

where *A*_*u*_, *A*_*Th*_, and *A*_*k*_ are the activity concentrations (Bq kg^-1^) of radionuclides in soil samples.

#### External and internal hazard indexes

The H_ex_ is a single index that measures the exposure to gamma radiation from ^232^Th,^238^U, and ^40^K radionuclides that are found naturally in soil. Using Eq. 4, the H_ex_ was computed (Akpanowo et al. 2020). It is noteworthy that the soil is commonly used as a construction material to build earthen houses in the study area.

Eq. 4 $${H}_{ex}=\frac{A_U}{370}+\frac{A_{Th}}{259}+\frac{A_K}{4810}$$

where *A*_*u*_, *A*_*Th*_, and *A*_*k*_ are the activity concentrations (Bq kg^-1^) of radionuclides in soil samples. The internal hazard index (*H*_*in*_) is a parameter used to assess the indoor radiological hazard from radionuclides in construction materials, including the exposure of radon and radon progeny originating from the radioactive decay of primordial radionuclides. The *H*_*in*_ was calculated with Eq. 5:

Eq. 5 $${H}_{in}=\frac{A_U}{185}+\frac{A_{Th}}{259}+\frac{A_K}{4810}$$

The external and internal hazard indices are acceptable (i.e., not exceeding the dose limit of 1 mSv y^-1^) when the calculated index is ≤ 1 (Purnama and Damayanti 2020).

#### Radioactivity level index for farmers

The radioactivity level index, noted by Iγ, has been proposed to estimate the magnitude of radiation risk from gamma emitting radionuclides in soil. It was estimated using Eq. 6 (Purnama and Damayanti 2020).

Eq. 6 $${I}_{\upgamma}=\frac{A_U}{150}+\frac{A_{Th}}{100}+\frac{A_U}{1500}$$

A_U_, A_Th_, and A_K_ are the activity concentrations (Bq kg^-1^) in soils of ^238^U, ^232^Th, and ^40^K, respectively. Radiation safety is considered acceptable if Iγ ≤1.

#### Absorbed dose rate

The absorbed dose rate by human beings (*D*) from exposure to external gamma radiation as a result of radioactive decay of ^238^U, ^232^Th, and ^40^K contained in soils was estimated using the activity-to-dose conversion factors (from Bq kg^-1^ in soils to nGy h^-1^ absorbed dose) and the activity concentrations A_U_, A_Th_, and A_K_ determined in soil samples by gamma spectrometry. The activity-to-dose conversion factors used were 0.462 (^238^U), 0.621 (^232^Th), and 0.0417 (^40^K). The absorbed dose rate was calculated using Eq. 7 (UNSCEAR 2008):

Eq. 7 $$D\ \left({nGyh}^{-1}\right)=0.462\ {A}_U+0.462{A}_{Th}+0.0417{A}_K$$

where *D* is the absorbed dose rate (nGy h^-1^) and *A*_*U*_, *A*_*Th*_, and *A*_*K*_ are the activity concentrations (Bq kg^-1^) of ^238^U, ^232^Th, and ^40^K in soil samples.

#### Annual effective dose for tobacco farmers from tobacco leaves

Through the root uptake of radionuclides from soils, tobacco plants can become an additional above ground reservoir of radionuclides. Therefore, we hypothesized that tobacco plantations (i.e., the plant biomass) might be a source of external radiation to farmers and workers of the plantations, and the relevant radiological indexes were calculated as follows.

Applying the ICRP activity-to-dose conversion factors (Sievert per gray (Sv/ Gy)), occupancy factors for indoor and outdoor (0.8 and 0.2, respectively), the time of a year (hours per year (h/y)), and the absorbed dose rate D (nGy h^-1^), the yearly effective dose was calculated (UNSCEAR 2008). The annual effective dose to tobacco farm workers outdoors (AED_o_) and indoors (AED_i_) were calculated using Eq. 8 and Eq. 9 (Taskin et al. 2009; Akpanowo et al. 2020):

Eq. 8 $$\textrm{For}\ \textrm{outdoor}:\textrm{AEDo}\Big(\left(\mu Sv\ {y}^{-1}\right)=D\left( nGy\ {h}^{-1}\right)\times 8760h\ {y}^{-1}\times 0.8\times 0.7\left( Sv\ {Gy}^{-1}\right)\times {10}^{-3}$$

Eq. 9 $$\textrm{For}\ \textrm{indoor}:\textrm{AEDi}\Big(\left(\mu Sv\ {y}^{-1}\right)=D\left( nGy\ {h}^{-1}\right)\times 8760h\ {y}^{-1}v\times 0.2\times 0.7\left( Sv\ {Gy}^{-1}\right)\times {10}^{-3}$$

The total annual effective dose AED is the sum of AEDo and AEDi. It should not exceed an annual dose limit of 1 mSv y^-1^ to be considered acceptable for the general public.

#### Annual effective dose from tobacco inhalation

Tobacco can enter the human lungs through snuffing or smoking. A distinction was made as follows: the calculation of radiation dose from tobacco snuffing assumes that the gamma-emitting radionuclides present in tobacco reach the lungs. In contrast, in the case of smoking, only a fraction of the present radionuclides reach the lungs, and other fractions escape through the smoke main stream into the air or are retained by the cigarette filter.

The annual effective dose of tobacco leaf consumption by snuffing was calculated using Eq. 10.

Eq. 10 $${E}_{\textrm{snuffing}}=A\left( Bq\ {kg}^{-1}\ \right)\times M(kg)\times {D}_{cf}\left( Sv\ {Bq}^{-1}\right)$$

where *A* is the activity concentration of individual radionuclides in tobacco leaves, *M* is the total mass of the snuffed tobacco in one year, and *D*_*cf*_ is the activity-to-dose conversion coefficient (Sv Bq^-1^).

The annual effective dose inhaled from tobacco/cigarette smoke was calculated using Eq. 11.

Eq. 11 $${E}_{\textrm{smoking}}=0.75\times 0.5\times A\left( Bq{kg}^{-1}\right)\times M\left( kg\ {y}^{-1}\right)\times {D}_{cf}\left( Sv\ {Bq}^{-1}\right)$$

It was estimated and assumed that about 75% of the radionuclides present in tobacco and tobacco products (cigars, cigarettes, etc.) pass into smoke. However, only 50% of that smoke reaches the lungs, while the other 50% is released into the air (side smoke) (UNSCEAR 2008). Therefore, the smokers and snuffers inhalation dose conversion coefficients for individual radionuclides used were 2.9 10^-6^ Sv Bq^-1^ for ^238^U, 4.5 10^-5^ Sv Bq^-1^ Th^232^, and 2.1 10^-9^ Sv Bq^-1^ for ^40^K.

The annual effective dose from tobacco smoking and snuffing was calculated using local statistics available for cigarette consumption in Tanzania (GATS 2018). The calculations were based on the consumption of 8.5 cigarettes per day per smoker/snuffer (GATS 2018). The average weight of tobacco rolled by the smoker/snuffer in a piece of paper, banana leaf, maize leaf (traditionally known as “roll-your-own tobacco”), or proper cigarette paper is approximately 1.3 grams. Therefore, the weight of tobacco consumed annually in Tanzania was calculated considering the weight of tobacco in a cigarette multiplied by the number of cigarettes smoked per day (1.3 g × 8.5 = 11.05 g per day), which yields the annual mass of tobacco consumed of approximately 4 kg per person (11.05 g per day per person × 365 days per year = 4.033 kg y^-1^). Unfortunately, the information on partitioning the amount of tobacco consumed as smoked or snuffed per year was not available, and it was assumed that the amount of tobacco snuffed is equal to the amount smoked. This information was used in Eq. 10 and Eq. 11 to estimate the effective dose for tobacco consumers through inhalation.

#### Excess lifetime cancer risk (ELCR) for tobacco consumers

Adoption of the linear non-threshold (LNT) dose-response model proposed by the International Commission for Radiological Protection (ICRP) implies that low radiation doses from naturally occurring radioactive materials can potentially induce cancer in exposed individuals. Therefore, the risk of developing cancer from low doses over the person’s lifetime or the excess lifetime cancer risk (ELCR) for smoking and snuffing was calculated using Eq. 12 and Eq. 13 (Kadhim and Ridha 2019).

Eq. 12 $${\textrm{ELCR}}_{\textrm{smoking}}={A}_{ls}\times {E}_{\textrm{smoking}}\times 0.05{Sv}^{-1}$$

Eq. 13 $${\textrm{ELCR}}_{\textrm{snuffing}}={A}_{ls}\times {E}_{\textrm{snuffing}}\times 0.05{Sv}^{-1}$$

where ELCR is the excess lifetime cancer risk, *A*_*ls*_ is the average life expectancy (75 years) for snuffers and smokers, 0.05 Sv^-1^ is the mortality risk coefficient for inhalation, and *E*_smoking_ and *E*_snuffing_ are the annual effective doses from smoking and snuffing, respectively.

According to United Nations Commodity Trade Statistics Database (UN Comtrade) 2022, tobacco and tobacco products are consumed in the producing nations (Kenya, Tanzania, and Uganda), as well as other African nations (Burundi, Malawi, Rwanda, and South Africa), with average life spans of 65 years. Therefore, the ELCR from snuffed and smoked tobacco and tobacco products was calculated using an *A*_*ls*_ value of 65 years, the average life expectancy across the three countries.

### Statistical analyses

Following descriptive statistical evaluation using the Shapiro-Wilk normality test, the distribution of all data was determined to be normally distributed (*p* < 0.05). Then, further analysis was done using Tukey Honestly Significant difference (HSD) to show the differences between the means. Finally, the degree of the relationships between the radionuclide’s concentration (^238^U, ^232^Th, and ^40^K) and soil physical-chemical properties was determined using Pearson’s correlation analysis (*p* < 0.05). The statistical analyses were performed using the STATISTICA (8^th^ Edition) software.

## Results and discussion

### One-year experimental results

The activity concentrations of ^238^U, ^232^Th, and ^40^K in the NPK fertilizers used in the one-year experimental plots are indicated in Table 2. These fertilizer brands are also the most commonly used in tobacco plantations in Kenya, Tanzania, and Uganda. The results show that the GLT fertilizer had the highest ^232^Th concentration (687.7 ± 1.5 Bq kg^-1^) and the highest ^238^U concentration (3,216.3 ± 21.7 Bq kg ^-1^). The high radioactivity of the GLT fertilizer is associated with the radioactive nature of the local phosphate ore, which serves as the primary raw material in fertilizer production (Mwalongo et al. 2022). Potassium is one of the macronutrients present in the fertilizer. It mainly comprises the stable isotope ^39^K, but it always contains the radioactive isotope ^40^K in a fixed proportion, and ^40^K makes up about 0.012% (120 mg kg^-1^) of the elemental potassium.

**Table 2:** Activity concentrations of radionuclides (Bq kg ^-1^ dry weight) in NPK fertilizers used in this study and their leaf yield

Fertilizers trade name	Activity concentration (Bq kg ^-1^)			Dry leaf yield (kg ha^-1^)
	^238^U	^232^Th	^40^K	
Yaramila Blended (YB)	514.6 ± 4.2b	37.3 ± 0.9b	2,179.7 ± 102.7a	2,144.18 ± 77.15a
Yaramila Compound (YC)	198.6 ± 6.1c	26.3 ± 0.3c	1,566.7 ± 98.5b	1995.25 ± 65.27b
Golden Leaf Tobacco (GLT)	3,216.3 ± 21.7a	687.7 ± 1.5a	760.7 ± 34.8c	1515.43 ± 66.77c
Unfertilized				478.70 ± 58.63d

In Table 2, the letters indicate the Tukey significant difference among the average concentrations of ^232^Th, ^238^U, and ^40^K across the column. The Tukey’s multiple pairwise honestly significant (THSD) statistical test, the column with different letter(s) are significantly different at *p* ≤ 0.0 5 confidence level. Meaning of the letters: “a” is very highly significant; “b” is very significant; “c” is significant; “d” is non-significant

Compared to the other fertilizers, YC showed much lower amounts of ^232^Th (26.3 ± 0.3 Bq kg^-1^) and ^238^U (198.6 ± 6.1 Bq kg^-1^), indicating that the source material for YC phosphate fertilizer may have low radioactivity levels. Despite the low-level radioactive detection, the product (YC) is sufficiently suitable for use as fertilizers for tobacco. According to the much greater ^40^K radionuclide concentration (1,566.7 ± 98.5 Bq kg^-1^) found in YB, the raw materials stable potassium (^39^K) may include the radioactive ^40^K (Anguani 2017). The crop leaf yield (kg ha^-1^) was affected by fertilizer application. Applying YB and YC increased leaf yield and ^40^K-content significantly compared to leaves grown using GLT (Table 2). The increase in tobacco leaf ^40^K was due to the applied good quality NPK fertilizers.

**Fig. 3:**
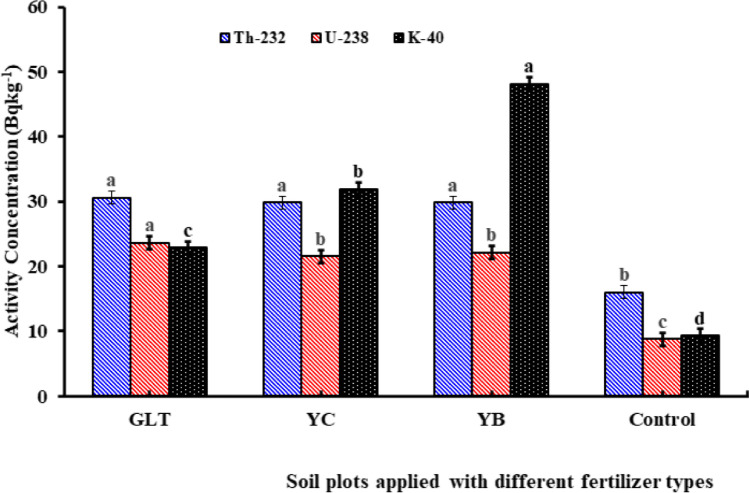
Activity concentrations of ^232^Th, ^238^U, and ^40^K in soils after application of different NPK fertilizers (GLT, YC, and YB) in the one-year experimental plots

Results from the one-year experimental plots are shown in Fig. 3. The concentrations of the ^238^U, ^232^Th, and ^40^K activity in NPK-treated soils were significantly (*p* < 0.05) higher than the soil from the control plot where no fertilizers were applied (Fig. 3). The results also show that the soil treated with YB displayed significantly (*p* < 0.05) higher ^40^K activity concentration than soil from the other plots. High ^238^U levels in the soil are assumed to be caused by the high ^238^U content of the phosphate rock used in fertilizer production. The soil treated with GLT fertilizer contained significantly (*p* < 0.05) higher ^238^U activity concentration (30.57 ± 1.3 Bq kg^-1^) if compared with the other plots.

There were no significant differences in activity concentrations of ^238^U and ^232^Th in soils treated with YB and YC fertilizers (Fig. 3). Low ^238^U and ^232^Th in YB and YC fertilizer is attributed to low ^238^U and ^232^Th in the source rock or the radionuclides could have been washed out by rainfall. Furthermore, the Tukey’s Honestly Significant Difference (THSD) post hoc test revealed that the activity concentrations of ^238^U, ^232^Th, and ^40^K after fertilizer application did not vary significantly with soil depth, i.e., the tillage of the soil distributed the fertilizers in the 30 cm topsoil layer.

In Fig. 3, the letters indicate the Tukey significant difference among the average concentrations of ^232^Th, ^238^U, and ^40^K across the column. In the Tukey’s multiple pairwise honestly significant (THSD) statistical test, the different letter(s) on each similar bar chart are significantly different at *p* ≤ 0.0 5 confidence level.

**Fig. 4:**
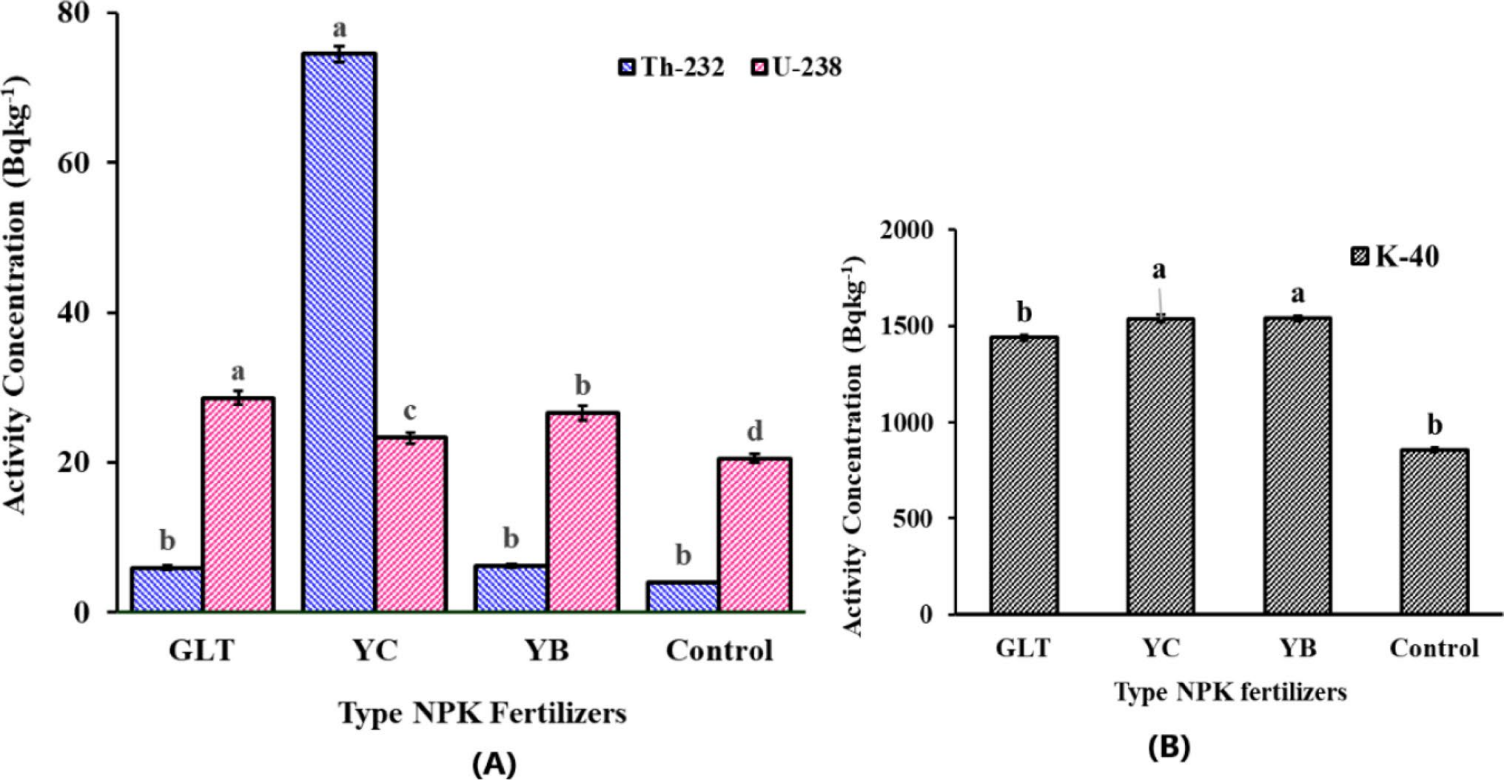
The activity concentration for ^238^U, ^232^Th (A), and ^40^K (B) in tobacco leaves after NPK fertilizer application in the one-year experiment

The activity concentrations of ^232^Th, ^238^U, and ^40^K in tobacco leaves from the one-year experimental plots are shown in Fig. 4. The activity concentrations of ^232^Th, ^238^U, and ^40^K in tobacco leaves from plots treated with NPK fertilizers were in the ranges of 5.97 ± 0.36 Bq kg^-1^ (GLT) to 74.45 ± 2.8 Bq kg^-1^ (YC) for ^232^Th, 24.56 ± 0.78 Bq kg^-1^ (YB) to 28.58 ± 1.60 Bq kg^-1^ (GLT) for ^238^U, and 1438.79 ± 12.1 Bq kg^-1^ (GLT) to 1539.38 ± 52.6 Bq kg^-1^ (YB) for ^40^K. The results showed that the leaves of tobacco plants grown with NPK fertilizers displayed significantly higher ^238^U concentrations than those of the control plants. The tobacco leaves showed a similar concentration of ^232^Th for YB and GLT but a statistically higher ^232^Th concentration for the YC fertilizer.

The ^238^U uptake was significantly highest for tobacco leaves applied with GLT, followed by YC and YB. Conversely, the unfertilized tobacco leaves (control) had the significantly lowest uranium content (see Fig. 4). It is also noteworthy that the fertilized tobacco leaves showed considerably higher radionuclide concentrations than tobacco leaves analyzed elsewhere (Söğüt et al. 2014; Landsberger et al. 2015).

The activity concentrations of ^40^K in tobacco leaves were significantly higher than those of ^238^U and ^232^Th (Fig. 4) in plots treated with YB and YC fertilizers. The higher concentration of ^40^K is likely due to the hyperabsorption of potassium by tobacco plants in comparison with other macronutrients (Çalişkan and Çalişkan 2018; Eke and Ishfaq 2021). The activity concentrations of ^232^Th were significantly higher in tobacco leaves from plots treated with YC fertilizer compared with GLT, YB, and the control group. Except for the plot treated with YB fertilizer, the activity concentrations of ^238^U and ^232^Th in soils were still lower than the worldwide average concentrations of ^238^U (35 Bq kg^−1^) and ^232^Th (30 Bq kg^−1^) in soils reported by the United Nations Scientific Committee on Atomic Radiations estimates (UNSCEAR 2008). These experimental results show that when NPK fertilizers with high ^238^U, ^232^Th, and ^40^K concentrations are applied, a transfer of these radionuclides from the soil to the tobacco plant is taking place.

The physical and chemical properties of soil from the one-year experimental plots were determined, and the relationship between ^232^Th, ^238^U, and ^40^K activity concentrations and soil physical-chemical properties was investigated. The results of a simple correlation matrix between the radionuclide concentrations and the physical-chemical properties is provided in Table 3. The correlation matrix revealed a moderately positive relationship between ^232^Th and the soil sand fraction, and a moderately negative relationship with electrical conductivity, silt and clay fractions in soil, and an extremely weak relationship with soil pH. Thus, a strong positive correlation of ^238^U with sandy soils could also reflect the ability of tobacco plant preference in sandy soils (Lisuma et al. 2020, 2021).

**Table 3:** Pearson’s correlation coefficients between ^232^Th, ^238^U, and ^40^K concentrations with some soil physical-chemical parameters for the one-year experiment

Parameter	^232^Th	^238^U	^40^K	pH	EC	OC	Sand	Silt	Clay
^**232**^**Th**	1								
^**238**^**U**	0.66 ns	1							
^**40**^**K**	-0.99^*^	-0.55 ns	1						
**pH**	0.91^*^	0.44 ns	-0.93^*^	1					
**EC**	0.98^*^	0.54 ns	-0,94^*^	0.94^*^	1				
**OM**	0.97^*^	0.51 ns	-0.99^*^	0.97^***^	0.99^*^	1			
**Sand**	-0.84^*^	-0.56 ns	0.82^*^	-0.7^*^	-0.81^*^	-0.78^*^	1		
**Silt**	0.94^*^	0.52 ns	-0.96^*^	0.92^*^	0.97^*^	0.97^*^	-0.71^*^	1	
**Clay**	0.76^*^	0.38 ns	-0.77^*^	0.78^*^	0.74^*^	0.76^*^	-0.83^*^	0.59 ns	1

^*^Significant at *p* ≤ 0.05, ^**^p ≤ 0.01, and ^***^p ≤ 0. 001; *ns*, not significantly differences

The ^238^U displayed a strong positive correlation with sandy soil and a strong negative correlation with EC, silt and clay in the soil, and a moderate positive correlation with soil pH and organic carbon content. Furthermore, the ^238^U concentration displayed a strong positive correlation with ^232^Th and is moderately correlated with the ^40^K concentration.

Potassium (^40^K) displayed a strong negative correlation with the soil pH, a moderately negative relationship with electrical conductivity, silt and clay fractions in soil, and an extremely weak relationship with organic carbon.

It has been reported that the behavior of uranium and other radionuclides in tropical soils depends upon radioelement solubility, soil pH, redox potential, cation exchange capacity (CEC), organic matter content, and soil texture (sandy, silted, and loamy) (Ribeiro et al. 2018; De Souza Braz et al. 2021). Similarly, according to Hu et al. (2020), soil physicochemical characteristics such as pH for sunflowers and peas showed the highest uranium uptake at pH 3–5. Another factor that affects uranium uptake by plants is the presence of carbonates and phosphates in the soil that have the propensity to interact with uranium to create complexes, which affect the absorption of uranium by plants (Sokolik et al. 2020; Tagami and Uchida 2020).

### Ten-year NPK fertilizer application experiment

**Fig. 5:**
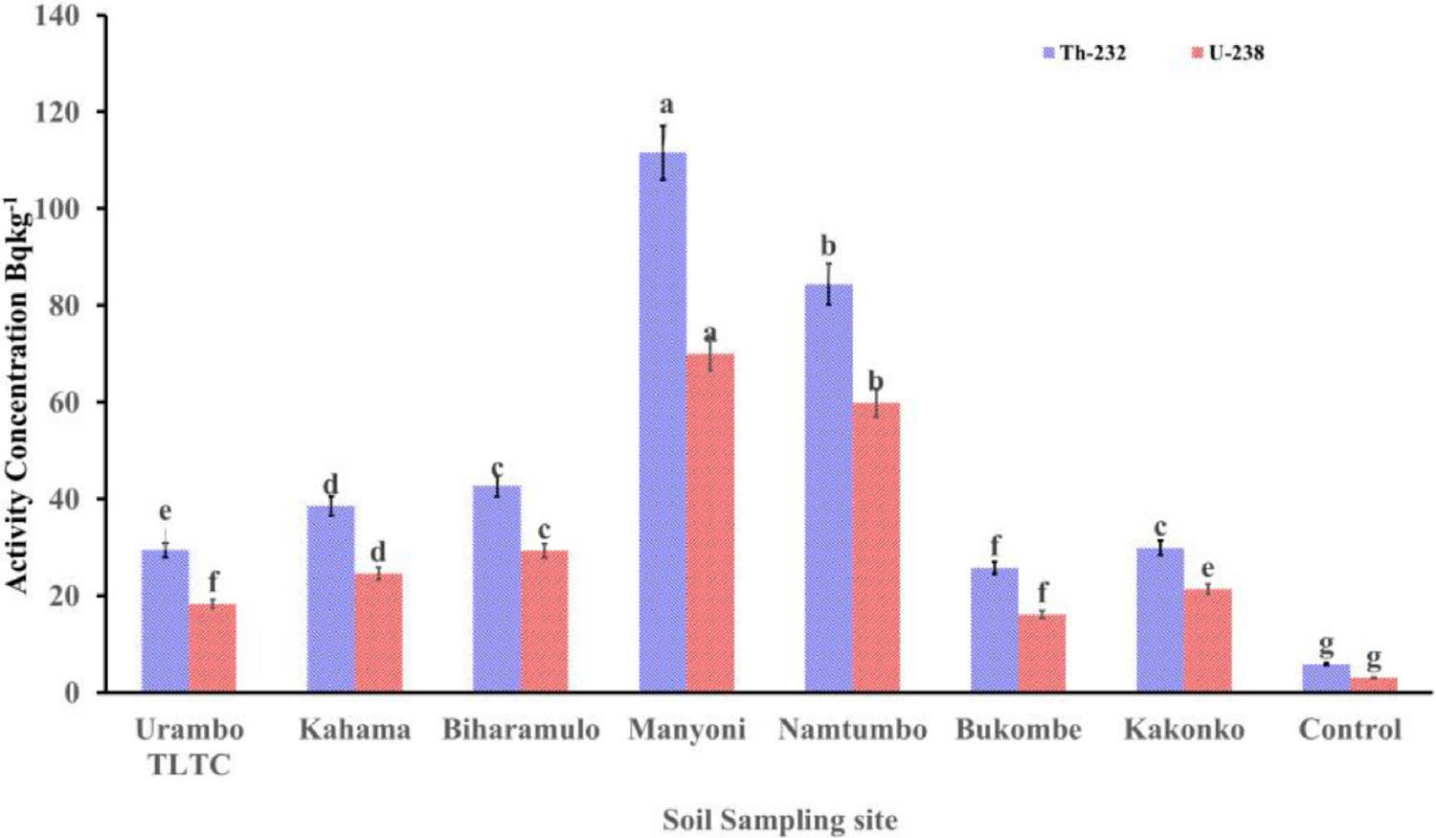
Concentration of ^232^Th and ^238^U in agricultural soils after ten-year application of NPK fertilizers application

**Fig. 6:**
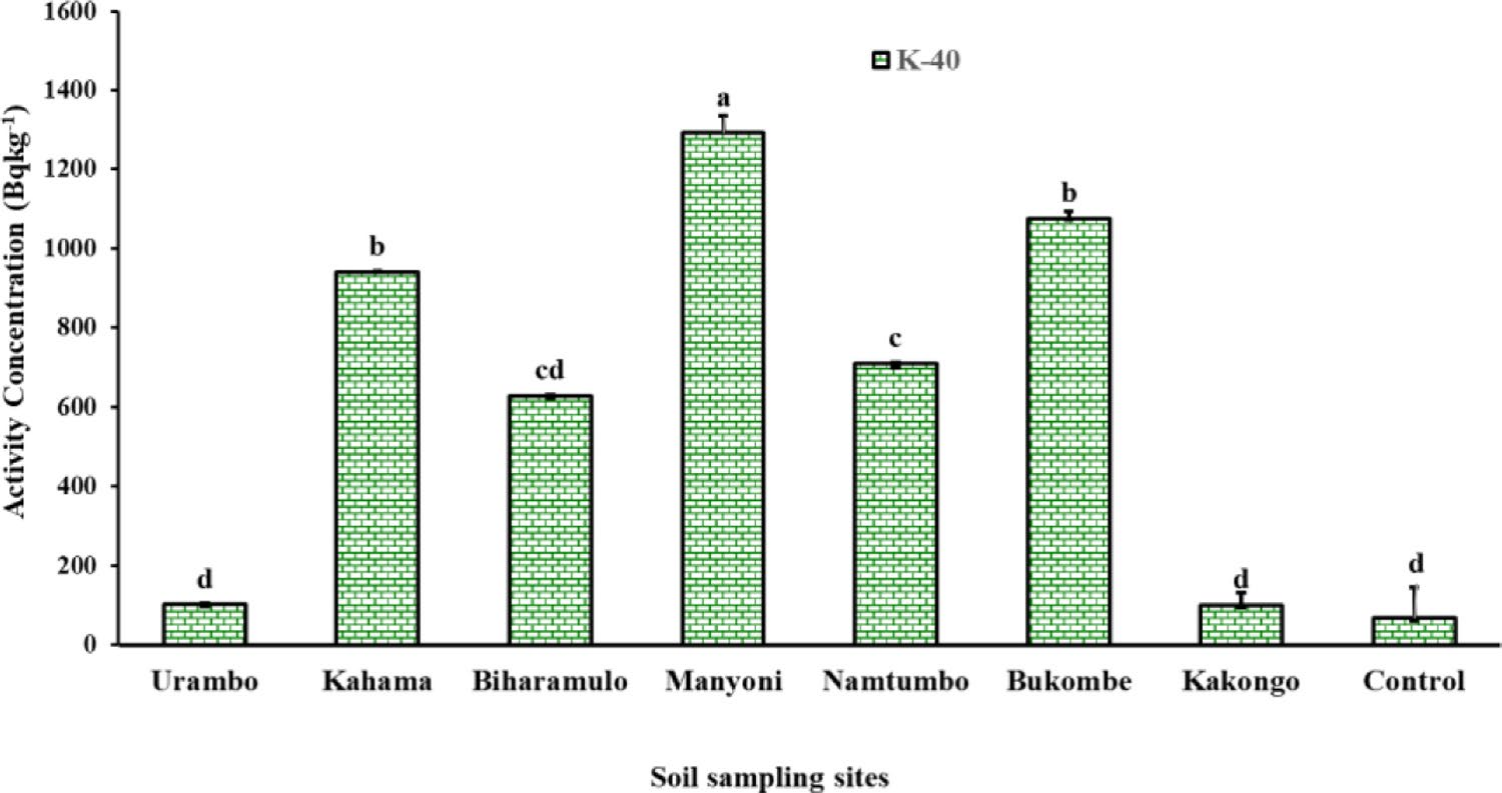
Concentration of ^40^K in agricultural soils after ten-year application of NPK fertilizers

The results showed that the concentration of ^232^Th, ^238^U, and ^40^K in agricultural soils used in this experiment was significantly higher (*p* < 0.05) after ten years of annual application of phosphate fertilizers than the control samples (no application of fertilizer) (Figs. 5 and 6).

After the annual application of NPK fertilizers for ten years, the activity concentrations of ^232^Th varied from 25.76 ± 0.9 Bq kg^-1^ (Bukombe district) to 111.58 ± 6.8 Bq kg^-1^ (Manyoni District). The activity concentrations of ^40^K varied significantly from 100.41 ± 7.5 (Bukombe district) to 1074.79 ± 86.0 Bq kg^-1^ (Bukombe District). The activity concentrations of ^238^U ranged from 16.1 ± 0.6 Bq kg^-1^ (Bukombe district) to 69.99 ± 2.8 Bq kg^-1^ (Manyoni District).

The higher ^238^U concentration in Manyoni was related to the prolonged phosphate fertilizer application, but the naturally occurring uranium in soils in this area is also slightly above average (Kasoga et al. 2015). Indeed, the Manyoni region hosts some surficial uranium occurrences, which may have contributed to the higher radioactivity in local agriculture soils if compared to other farms (IAEA/NEA 2020). The activity concentrations of ^238^U, ^232^Th, and ^40^K in the agricultural soils are shown in Figs. 5 and 6.

The results indicate that ^232^Th was not detected in tobacco leaves from the Biharamulo and Bukombe farms meaning that the value was below the method’s detection limit of 1.56 ± 0.12 Bq kg^-1^. Tobacco leaves from Kahama (33.73 ± 1.60 Bq kg^-1^) showed the highest ^232^Th concentration, followed by those from the Urambo farm (26.02 ± 1.50 Bq kg^-1^), Kakonko (15.73 ± 1.42 Bq kg^-1^), Manyoni (12.83 ± 1.03 Bq kg^-1^), and Namtumbo (12.43 ± 0.76 Bq kg^-1^). The range of ^232^Th concentrations in tobacco leaves reported in this study agrees well with those reported by Mkhaiber et al. (2020), who assessed the presence of ^232^Th in tobacco leaves from the UAE, France, Iraq, Armenia, Türkiye, and South Korea and reported ^232^Th concentrations ranging from 4.8 Bq kg^-1^ to 36.0 Bq kg^-1^.

**Fig. 7:**
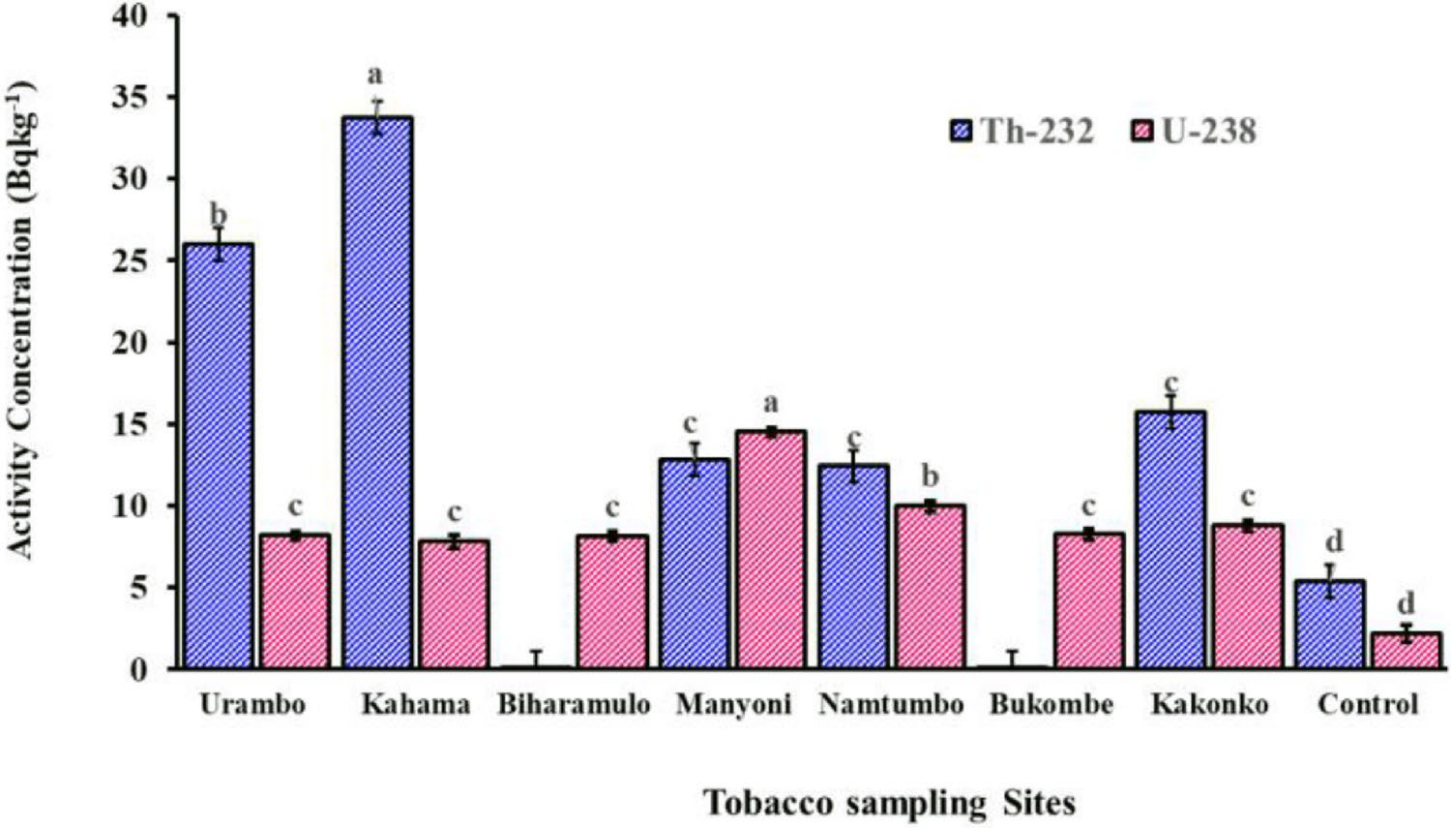
Activity concentrations of ^232^Th and ^238^U in tobacco leaves grown in experimental farms with soils fertilized in the previous ten years

The concentrations of ^238^U in the same tobacco leaves (Fig. 7) ranged from 7.83 ± 0.1 Bq kg^-1^ to 14.52 ± 0.62 Bq kg^-1^. Tobacco leaves from Manyoni showed the highest concentrations of ^238^U compared to other studied areas, followed by Namtumbo (9.98 ± 0.51). The concentration did not significantly differ for Kakonko (8.80 ± 0.45), Bukombe (8.26 ± 0.49), Urambo farm (8.21 ± 0.65), Biharamulo (8.14 ± 0.37), and Kahama (7.83 ± 0.86). The presence of surficial and sedimentary uranium mineralization in Manyoni and Namtumbo (Banzi et al. 2015; Ngulimi and Ishiga 2016), respectively, may have influenced the higher ^238^U concentrations in the soils which can then increase uptake by tobacco leaves. The concentrations of ^238^U in tobacco leaves from the Bukombe and Biharamulo sampling sites were not detected (below the detection limit).

The results show that the concentration of ^232^Th decreased from Kahama down to Urambo, Kakonko, Manyoni, and Namtumbo (located in the southern part of Tanzania). The ^232^Th decreasing trend may be attributed to the variation of the physical-chemical properties of the soils. Thus, an increase in acidity from Kahama to Namtumbo influenced the increase of ^238^Th. A similar observation was reported by Gupta et al. (2020), whose results showed an increase of Th in more acidic soils and U decreasing in acidic soils.

**Fig. 8:**
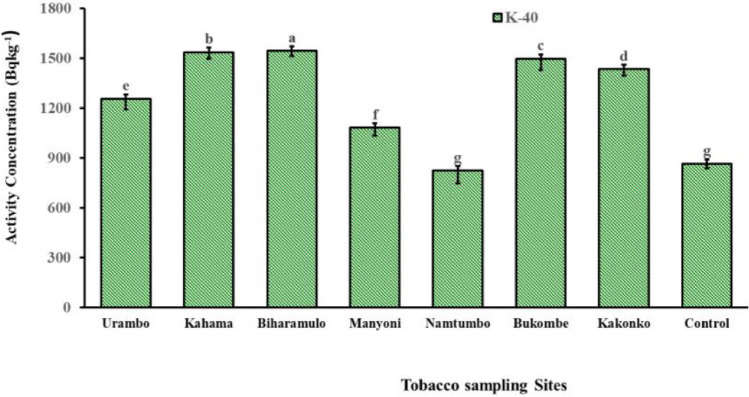
Activity concentrations of ^40^K in tobacco leaves grown in experimental farms with soils fertilized in the previous ten years

The concentration of ^40^K (Fig. 8) was significantly higher in tobacco leaves from Biharamulo (1,545.30 ± 58.87 Bq kg^-1^) and Kahama (1,538.14 ± 32.3 Bq kg^-1^) followed by Bukombe (1,497.83 ± 78.89 Bq kg^-1^), Kakonko (1,434.3 ± 78.7 Bq kg^-1^), Urambo (1,255.1 ± 60.9 Bq kg^-1^), Manyoni (1,081.8 ± 46.6 Bq kg^-1^), and Namtumbo (823.9 ± 75.9 Bq kg^-1^). However, the ^40^K concentration of Namtumbo was statistically similar to the control sample.

The physical-chemical soil factors influence the concentration of ^238^U, ^232^Th, and ^40^K in the soil. Studies have shown that the geology and soil structure influence environmental concentration of radionuclides of natural origin (UNSCEAR 2008). The concentration of ^238^U, ^232^Th, and ^40^K can also be influenced by soil weathering, sediment accretion, sorption, leaching, and circulation of groundwater and surface water (Gupta and Walther 2019; Ratnikov et al. 2020). All these factors certainly played a role in the varying radionuclide concentrations among regions, as found in this investigation. In the ten (10) year’s experiment, it was, however, impossible to determine the exact role of each factor. Nevertheless, it was found that there is a strong and positive correlation between ^238^U and ^232^Th, and both displayed a strong negative correlation with organic matter in the soil. Furthermore, the sandy soil was positively correlated with ^238^U and ^232^Th and negatively correlated with organic matter and silt, as indicated in Table 4.

**Table 4:** Pearson’s correlation coefficients between ^232^Th, ^238^U, and ^40^K concentrations with some soil physical-chemical parameters for the ten years experiment

	^232^Th	^238^U	^40^K	pH	EC	OC	Sand	Silt	Clay
^232^Th	1								
^238^U	-0.24 ns	1							
^40^K	-0.16 ns	-0.13 ns	1						
pH	0.61^*^	-0.61^*^	-0.30 ns	1					
EC	-0.43 ns	0.49 ns	0.28 ns	-0.94^*^	1				
OM	-0.76^*^	0.66^*^	0.27 ns	-0.83^*^	0.59^*^	1			
Sand	-0.42 ns	-0.38 ns	0.19 ns	-0.27 ns	0.30 ns	0.14 ns	1		
Silt	-0.38 ns	-0.36 ns	0.09 ns	-0.24 ns	0.28 ns	0.11 ns	0.99^*^	1	
Clay	-0.08 ns	-0.36 ns	0.40 ns	-0.06 ns	0.13 ns	-0.08 ns	0.47^*^	0.43 ns	1

^*^Significant at *p* ≤ 0.05, ^**^p ≤ 0.01, and ^***^p ≤ 0.001; *ns*, not significantly differences

### Investigation in selected tobacco farms in Kenya, Tanzania, and Uganda

The activity concentration of ^232^Th, ^238^U, and ^40^K was determined in the agricultural soils of farms in Kenya, Tanzania, and Uganda for the soil layer with 0–30 cm depth.

**Fig. 9:**
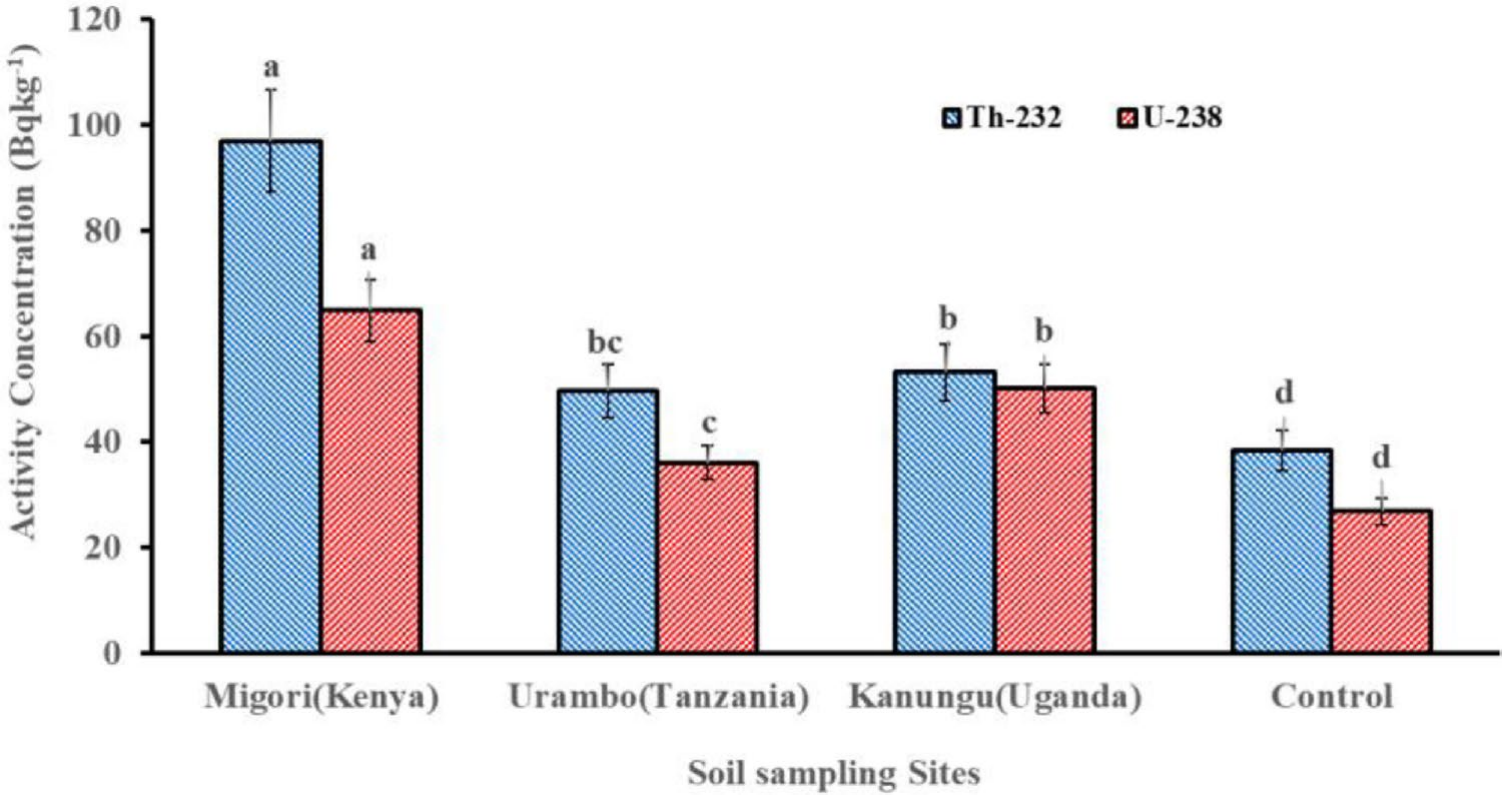
Activity concentrations of ^232^Th and ^238^U in soils from tobacco fields from Kenya, Tanzania, and Uganda

**Fig. 10:**
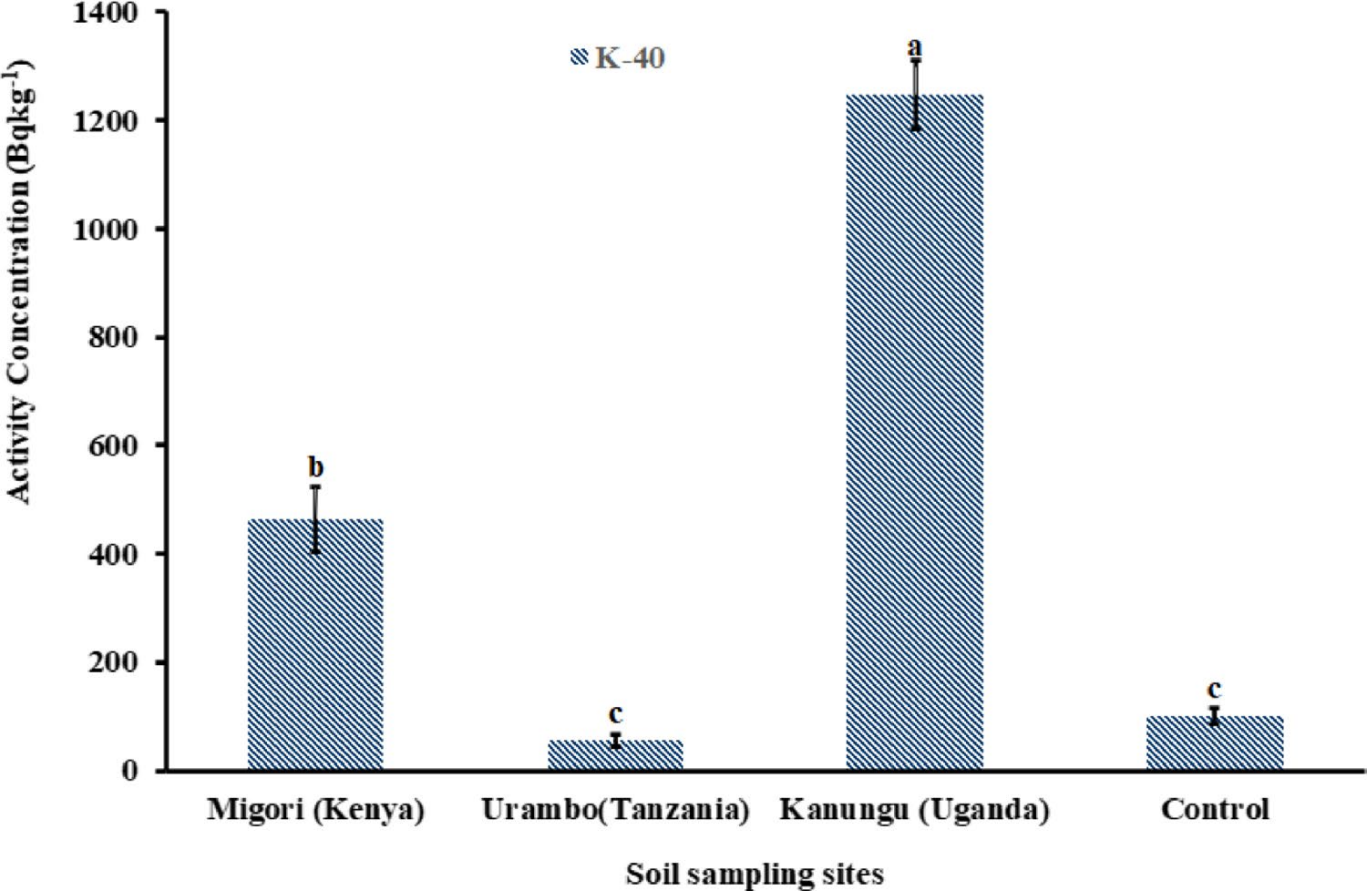
Activity concentrations of ^40^K in soils from tobacco fields from Kenya, Tanzania, and Uganda

The concentrations of ^232^Th, ^238^U, and ^40^K in soils were significantly higher (*p* < 0.05) than the control sample from the non-fertilized farm in Tabora, Tanzania (Figs. 9 and 10). Migori (Kenya) had a significantly higher ^238^U concentration, followed by Kanungu (Uganda), which showed the lowest value that was still significantly higher than the control group. A similar result trend was observed for ^232^Th (Fig. 9). However, for ^40^K, Kanungu (Uganda) had a significant concentration, followed by Migori (Kenya), and Urambo (Tanzania) had the lowest ^40^K concentration (Fig. 10), which was similar to the control group. The variations in natural radioactivity in soil depend on the unique natural radionuclides present at the sampling sites, the parent rock that formed the soil, and other forming variables (UNSCEAR 2008).

**Fig. 11:**
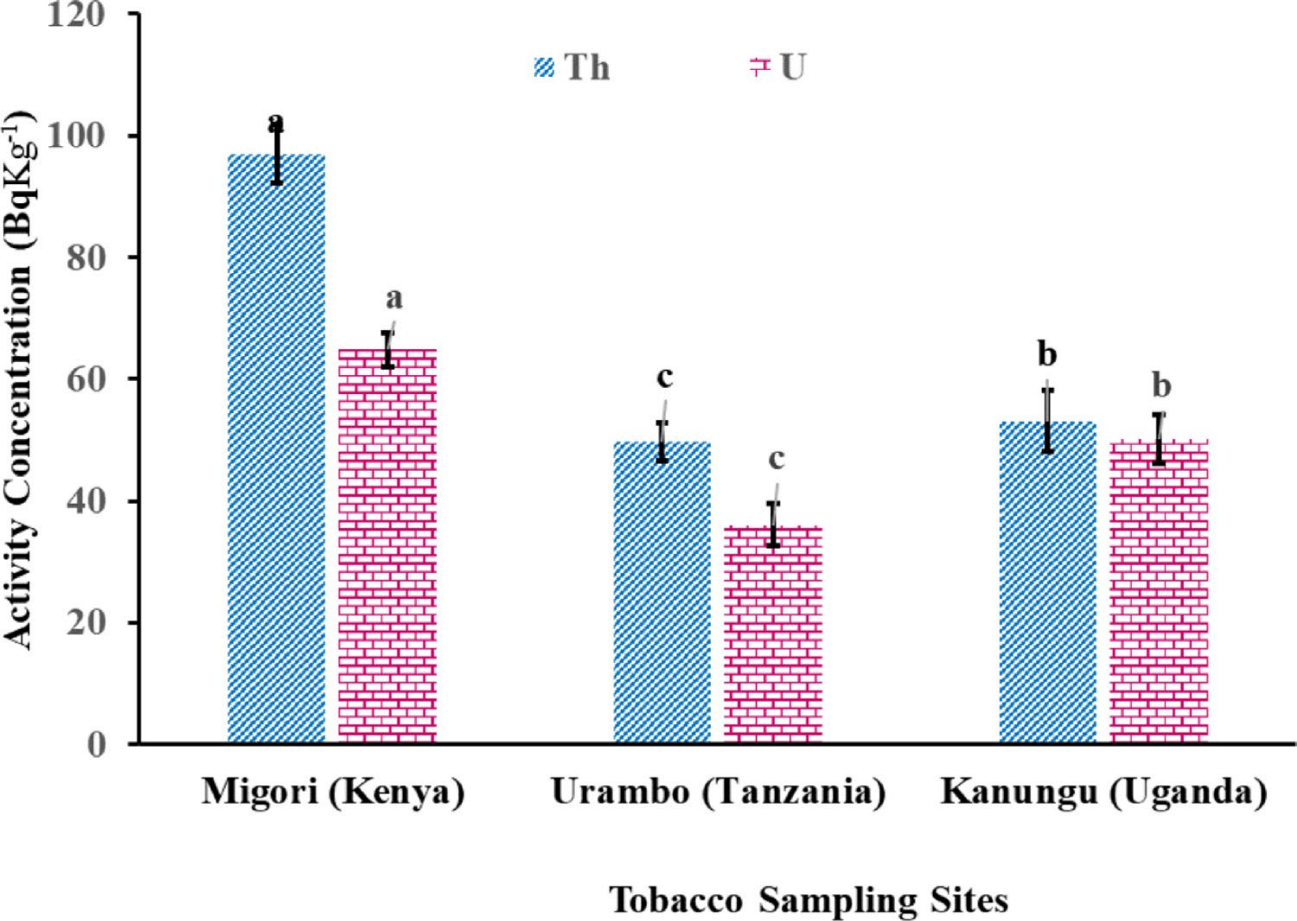
Activity concentrations of ^232^Th and ^238^U in tobacco leaves from Kenya, Tanzania, and Uganda

**Fig. 12:**
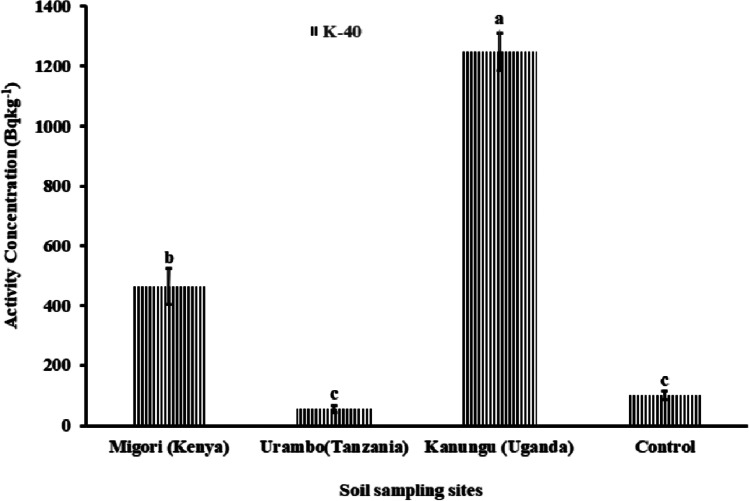
Concentration of ^40^K in tobacco leaves from selected tobacco farms in Tanzania, Kenya, and Uganda

The concentration of radionuclides in tobacco leaves from Kenya, Tanzania, and Uganda ranged from 49.6 ± 3.9 Bq kg^-1^ to 96.9 ± 8.4 Bq kg^-1^ for ^232^Th, 36.6 ± 3.2 Bq kg^-1^ to 64.8 ± 5.8 Bq kg^-1^ for ^238^U, and 55.0 ± 4.5 Bq kg^-1^ to 1247.5 ± 87.8 Bq kg^-1^ for ^40^K as depicted in Figs. 11 and 12. Tobacco leaves from Migori (96.9 ± 4.8 Bq kg^-1^) displayed the highest ^232^Th concentrations, followed by those from Kanungu (53.1 ± 5.0 Bq kg^-1^), with the lowest concentrations measured in leaves from Urambo (49.6 ± 3.1 Bq kg^-1^). Samples from Migori also displayed the highest ^238^U concentration (64.8 ± 2.8 Bq kg^-1^) followed by those from Kanungu (50.1 ± 4.1 Bq kg^-1^), and the lowest concentrations were again measured in leaves from Urambo (36. 1 ± 3.4 Bq kg^-1^).

Tobacco leaves from Kanungu showed the highest ^40^K concentration (1247.5 ± 24.0 Bq kg^-1^), followed by those from Migori (464.5 ± 15.8 Bq kg^-1^) and Urambo (55.1 ± 4.3 Bq kg^-1^) as shown in Fig. 12.

In these three countries, the radionuclide concentrations in tobacco leaves were globally higher than in the 1-year and 10-year experiments for ^238^U and ^232^Th. However, the ^40^K concentrations were even lower than that of the control sample.

The Pearson’s correction matrix on the association between ^232^Th, ^238^U, and ^40^K concentrations and the physical-chemical parameters for agricultural soils of Kenya, Tanzania, and Uganda are presented in Table 5. The results showed a negative association of sand and silt with that of OM, indicating that the OM content was higher in clayey soils. The negative association may be because sand and silt lose OM more rapidly than clayey soils through natural and anthropogenic processes (Arunrat et al. 2020). Another possible reason is that indigenous tobacco farmers uproot stalks from the field after harvest and burn them, thus reducing organic carbon from the decay of plant materials in soils (Lisuma et al. 2022b). This result is similar to that Lisuma et al. (2022a) reported earlier, who found that Tabora and Urambo soils are mainly composed of sand and silt and contain the lowest OM content.

**Table 5:** Pearson’s correlation coefficients between ^232^Th, ^238^U, and ^40^K dry weight concentrations with soil physical-chemical parameters at selected tobacco farms in Kenya, Tanzania, and Uganda

Parameter	pH	EC	OM	Sand	Silt	Clay	^232^Th	^238^U	^40^K
pH	1								
EC	0.42								
OM	-0.12	0.69^*^	1						
Sand	0.38	-0.49	-0.96^*^	1					
Silt	-0.05	0.63	0.97^*^	-0.92^*^	1				
Clay	-0.51	0.39	0.91^*^	-0.99^*^	0.87	1			
^232^Th	-0.41	-0.74^*^	-0.83^*^	0.68	-0.86	-0.57	1		
^238^U	-0.07	-0.7	0.97^*^	0.89	0.98^*^	0.82	0.93	1	
^40^K	0.94^*^	0.12	-0.45	0.66	-0.36	-0.76^*^	-0.1	0.26	1

^*^Significant at *p* ≤ 0.05, ^**^p ≤ 0.01, and ^***^p ≤ 0. 001; *ns*, not significantly differences

The results further indicate a strong positive association between ^238^U and OM. The association between ^238^U and OM results from soil OM reducing the water-soluble and mobile hexavalent uranium U(VI) to the insoluble tetravalent U(IV), and thus, over time, the U concentrations in soils increase (Sokolik et al. 2020). There was a positive correlation between the ^40^K concentration and the soil pH and a negative correlation between ^40^K and the clay soil fraction.

### Estimation of radiological hazards from agricultural soil and tobacco leaves

#### Radium equivalent activity of soils

The Ra_eq_ of agricultural soils from all study areas ranged from 68.17 ± 3.5 Bq kg^-1^ (Urambo) to 329.2 ± 12.8 Bq kg^-1^ (Manyoni) and is shown in Table 6. These values are below the Ra_eq_ value of 370 Bq kg^-1^, corresponding to delivering an annual dose of 1 mSv y^-1^ to public members (UNSCEAR 2008). The low Ra_eq_ implies that these soils can be used as a construction material, as is common in the investigated regions.

**Table 6:** Summary of calculated radiological hazard indices and excess lifetime cancer risk from agricultural soils in Tanzania, Kenya, and Uganda

Site	Ra_eq_ (Bq/kg)	*D* (nGyh^−1^)	AED (nSvy^−1^) outdoor	AED (nSvy^−1^) indoor	*H*_*ex*_	*H*_*in*_	ELCR_Outdoor_ (10^-6^)	ELCR_indoor_ (10^-6^)
**One-year experiment**								
GLT	68.69	30.14	37	150	0.19	0.25	0.13	0.52
YC	68.20	30.01	40	150	0.18	0.25	0.13	0.52
YB	**71.51**	33.59	40	160	0.19	0.25	0.14	0.58
**Ten-year experiment**								
Urambo	68.17	30.45	40	150	0.18	0.23	0.13	0.52
Kahama	152.11	73.87	90	360	0.41	0.48	0.32	1.27
Biharamulo	138.56	65.46	80	320	0.37	0.45	0.28	1.12
Manyoni	329.18	153.73	190	750	0.89	1.08	0.66	2.64
Namtumbo	235.17	108.24	130	530	0.64	0.80	0.46	1.86
Bukombe	135.70	67.83	80	330	0.37	0.41	0.29	1.16
Kakonko	70.38	31.45	40	150	0.19	0.24	0.14	0.54

#### Absorbed dose rate due to radioactivity in soils

The obtained results show that the absorbed dose rate from agricultural soils ranged from 32.38 nGy h^−1^ (Urambo) to 282.93 ± 14.67 (Bukombe) (see Table 6). The absorbed dose rate for the Urambo (32. 38 nGy h^−1^), the Yaramila compound experimental site (33.14 nGy h^−1^), Kakonko (40.5 nGy h^−1^), Kanungu (41.44 nGy h^−1^), and Migori (54.37 nGy h^−1^) were lower than the global average value of 60 nGy h^−1^(UNSCEAR 2008). Therefore, farmers and public members using these lands are exposed to low doses of gamma radiation from the agricultural soils. However, the absorbed dose rate at the Tanzania Leaf Tobacco Company Limited (TLTC) farm (Urambo, Tanzania 128.09 nGy h^−1^) was about twice the recommended value. On the other hand, agricultural fields such as Namtumbo (154.16 nGy h^−1^), the YB field experiment (167.86 nGy h^−1^), Kahama (175.26 nGy h^−1^), and Biharamulo (178.94 nGy h^−1^) were about three times higher than the worldwide average dose rate value. In comparison, Manyoni (231.89 nGy h^−1^) was four times higher than the recommended value, and Bukombe (282.93 nGy h^−1^) was five times the worldwide average global value.

#### The annual effective dose rate from radioactivity in soils

The calculated outdoor and indoor annual effective dose rates from soils in the tobacco plantations, as summarized in Table 6, ranged from 37.0 nSvh^-1^ (GLT experimental site) to 190.0 nSvh^-1^ (Manyoni) and 150 nSvh^-1^ (GLT, YC, Urambo, Kakonko) to 750 nSvh^-1^ (Manyoni), respectively. The annual effective dose rates were below the worldwide mean annual effective dose, which is about 500 μSv y^−1^. Moreover, the total annual effective doses have been found to vary depending on the geological parameters of the soil matrixes where tobacco plants are grown and are further influenced by anthropogenic activities such as applying mineral phosphate fertilizers (IAEA 2013; UNSCEAR 2008).

#### The external and internal hazard indexes

The results in Table 6 show that the *H*_*ex*_ and *H*_*in*_ of tobacco agricultural soil ranged from 0.18 (YC, Urambo) to 0.89 (Manyoni) and 0.23 (Urambo) to 1.08 (Manyoni), respectively. For the radiation hazard to be tolerable, it should be lower than 1, which is (mostly) the case here. An index result greater than 1 corresponds to an annual dose of more than 1 mSv y^-1^ (Imani et al. 2021). The results imply that if the soil is used as a building material, this practice can exceed the public annual effective dose constraint of 1 mSvy^-1^ (UNSCEAR 2008).

#### Tobacco leaf radium equivalent activity

Tobacco plants were assumed to constitute an above ground reservoir of ^232^Th, ^238^U, and ^40^K and thus a potential source of radiation exposure to human beings and surface soils. Accordingly, the values of Ra_eq_ with the lower value of 90.20 ± 4.6 Bq kg^-1^ in Namtumbo and the highest value of 174.50 Bq kg^-1^ at the Kahama sampling location were measured (Table 7). Compared to the worldwide average of 60 Bq kg^-1^ of Ra_eq_, all investigated tobacco plant leaves exceeded the worldwide average Ra_eq_ value for soils. Nevertheless, the equivalent radium activity ranged from 90.20 Bq kg^-1^ to 174.5 Bq kg^-1^, which is significantly lower than the UNSCEAR recommended combined activity concentration of 370 Bq kg^-1^ (UNSCEAR 2008; Mostafa et al. 2020), that again corresponds to an annual dose of 1 mSv y^-1^ for the general public. Therefore, the Ra_eq_ activity indicates that the tobacco crops do not pose a significant radiological risk to farm workers and relatives living nearby.

**Table 7:** The estimated radiometric parameters for the consumption of tobacco leaves from the 10 years experiment and traditional tobacco farms in Kenya, Tanzania, and Uganda

Site	Ra_eq_	AED (smoking) mSv y^-1^	AED (snuffing) mSv y^-1^	ELCR smoking (10^-3^)	ELCR snuffing (10^-3^)
10-year experiment:					
Urambo	142.06	1.89	5.04	7.08	18.89
Kahama	174.5	2.45	6.53	9.18	24.48
Biharamulo	127.27	0.01	0.03	0.05	0.12
Manyoni	115.16	0.93	2.49	3.50	9.33
Namtumbo	90.2	0.90	2.41	3.39	9.03
Bukombe	123.74	0.01	0.03	0.05	0.12
Kakonko	145.73	1.14	3.05	4.29	11.44
Traditional farms:					
Migori	106.7	0.87	2.31	3.25	8.67
Urambo TLTC	130.16	0.00	0.01	0.02	0.05
Kanungu	109.13	1.18	3.16	4.44	11.85
Worldwide average				0.29	0.29

Ra_eq_, AED (smoking), AED (snuffing), ELCR smoking, and ELCR snuffing were calculated using the respective equations given on the methodology section

#### Annual effective dose due to tobacco inhalation

The results shown in Table 7 indicate that the annual effective dose due to tobacco snuffing ranged from 0.01 to 6.53 mSv y^-1^, varying with the producing region. The tobacco leaves from the one-year experimental plots may lead to effective doses higher than the recommended safety limit value of 1 mSv y^-1^ if snuffed and may pose a severe radiological health hazard.

The annual effective dose equivalent for tobacco smoking ranged from 0.1 mSv y^-1^ to 2.45 mSv y^-1^, varying again with the tobacco producing regions. Tobacco smokers may receive an annual dose that is 50-194% higher than the recommended limit of 1.26 mSv y^-1^, the average annual global exposure to natural radiation sources from inhalation (UNSCEAR 2008; Zlobina et al. 2022). Therefore, snuffing and smoking tobacco can produce relatively high annual effective doses.

#### Excess lifetime cancer risk

The excess lifetime cancer risk (ELCR) values for tobacco snuffing ranged from 5.0 × 10^-5^ to 24.48 × 10^-3^ for snuffing and from 0.02 × 10^-3^ to 9.18 × 10^-3^ for smoking, respectively. Therefore, as shown in Table 7, tobacco snuffing and smoking increase the likelihood of cancer significantly if compared to the worldwide average exposure of non-smokers/snuffers.

## Conclusions

The activity concentrations of ^232^Th, ^238^U, and ^40^K were determined in the three NPK fertilizers used in growing tobacco crops in Kenya, Tanzania, and Uganda. The results showed that YB and GLT fertilizers had higher concentrations of ^232^Th, ^238^U, and ^40^K in soils than the worldwide average of ^238^U (35 Bq kg^−1^) and ^232^Th (30 Bq kg^−1^) in soils reported by UNSCEAR. In addition, a one-year controlled experiment revealed that NPK fertilizer application caused soil activity concentrations of ^232^Th, ^238^U, and ^40^K and subsequently increased tobacco leaves uptake compared to plots where no mineral fertilizers were applied (control).

The application of phosphate fertilizers for a longer-term (10 years experiment in this study) also increased concentrations of ^232^Th, ^238^U, and ^40^K in soils and harvested tobacco leaves compared to the unfertilized tobacco farms. Thus, selecting NPK fertilizer with a lower content of naturally occurring radionuclides could reduce agricultural soil contamination and reduce radionuclide soil-to-plant transfer and, therefore, reduce the radiological health impact through inhalation and ingestion to humans.

The results showed a clear relationship between the ^232^Th, ^238^U, and ^40^K concentrations and physical-chemical properties which varied according to the soil’s physical and chemical properties. However, the results also indicate that the concentration of the radionuclides was not correlated with the soil's physical-chemical properties.

Radiation hazard parameters such as the radium equivalent activity, gamma-absorbed dose, annual effective dose, external and internal hazard indexes, and gamma index to both soils and tobacco leaves to assess the radiological risk parameters for the public, in general, were determined in this work. The results show that all radiological health risk parameters resulted in values lower than the recommended safety limits. Hence, exposure to agricultural soil and tobacco crops fertilized with phosphate fertilizers poses radiological hazards to farm workers and members of the public that are within tolerable limits. Even using these soils as construction materials for rural houses (a common practice in the study area) does not pose a radiological threat to the population. Therefore, the current work recommends further studies to expand the knowledge on soil-to-plant radionuclide transfer and the role of fertilizers in such transfers in cereal crops since cereal crops are grown in rotation with tobacco plantations in the investigated regions. Even if the measured radioactivity levels are within tolerable limits, it is recommended that countries should use fertilizers with lower radioactivity levels for the conservation of soil quality and to reduce radionuclide concentrations in tobacco.

## References

[CR1] Abed N, Monsif M, Zakaly H (2022). Assessing the radiological risks associated with high natural radioactivity of microgranitic rocks: a case study in a northeastern desert of Egypt. Int J Environ Res Public Health.

[CR2] Akpanowo MA, Umaru I, Iyakwari S (2020). Determination of natural radioactivity levels and radiological hazards in environmental samples from artisanal mining sites of Anka, North-West Nigeria. Sci Afr.

[CR3] Anguani ME (2017) Activity levels of gamma ray emitting radionuclides in food crop samples in selected tobacco farming areas in Aura and Maracha districts. Dissertation. https://hdl.handle.net/20.500.12504/960

[CR4] Arunrat N, Kongsurakan P, Sereenonchai S, Hatano R (2020). Soil organic carbon in sandy paddy fields of Northeast Thailand: a review. Agronomy.

[CR5] Asaduzzaman K, Khandaker MU, Amin YM, Mahat R (2015). Uptake and distribution of natural radioactivity in rice from soil in north and west part of peninsular Malaysia for the estimation of ingestion dose to man. Ann Nucl Energy.

[CR6] Banzi FP, Kifanga LD, Bundala FM (2000). Natural radioactivity and radiation exposure at the Minjingu phosphate mine in Tanzania. J Radiol Prot.

[CR7] Banzi FP, Msaki PK, Mohammed NK (2015). Distribution of heavy metals in soils in the vicinity of the proposed Mkuju uranium mine in Tanzania. Environ Pollut.

[CR8] Bigalke M, Ulrich A, Rehmus A, Keller A (2017). Accumulation of cadmium and uranium in arable soils in Switzerland. Environ Pollut.

[CR9] Bigalke M, Imseng M, Schneider S (2020). Uranium budget and leaching in Swiss agricultural systems. Front Environ Sci.

[CR10] Çalişkan B, Çalişkan AC (2018). Potassium nutrition in plants and its interactions with other nutrients in hydroponic culture. Improvement of Quality in Fruits and Vegetables Through Hydroponic Nutrient Management.

[CR11] Carvalho FP, Oliveira JM (2006). Polonium in cigarette smoke and radiation exposure of lungs. Czechoslov J Phys.

[CR12] Chauhan P, Chauhan RP (2014). Measurement of fertilizers induced radioactivity in tobacco plants and elemental analysis using ICAP-AES. Radiat Meas.

[CR13] Chune MD, Takoy MM, Peter O (2022) Analysis of technical efficiency and its determinants among tobacco producers in Uganda: An application of data envelopment analysis. Int J Agric Res Innov Technol 12(1):24–29

[CR14] De Souza Braz AM, Da Costa ML, Ramos SJ (2021). Long term application of fertilizers in eastern amazon and effect on uranium and thorium levels in soils. Minerals.

[CR15] Eke C, Ishfaq M (2021). A study on the activity concentrations of natural radionuclides and annual effective dose values in some tobacco samples. Cumhuriyet Science J.

[CR16] GATS (2018) 2018 Tanzania global adult tobacco survey country report. https://www.nbs.go.tz/nbs/takwimu/tobacco/2018TanzaniaGATSReport.pdf

[CR17] Gupta DK, Walther C (2019). Uranium in plants and the environment.

[CR18] Gupta DK, Chatterjee S, Mitra A, Voronina A, Walther C (2020) Uranium and Plants: Elemental Translocation and Phytoremediation Approaches. In: Gupta D, Walther C (eds) Uranium in plants and the environment. Radionuclides and Heavy Metals in the Environment. Springer, Cham. 10.1007/978-3-030-14961-1_7

[CR19] Haneklaus N (2021) Unconventional uranium resources from phosphates. In: Encyclopedia of Nuclear Energy. Elsevier, pp 286–291. 10.1016/B978-0-12-819725-7.00152-5

[CR20] Haneklaus N, Sun Y, Bol R (2017). To extract, or not to extract uranium from phosphate rock, that is the question. Environ Sci Technol.

[CR21] Hu N, Zhang H, Ding D (2020). Influence of uranium speciation on plant uptake. Uranium in Plants and the Environment.

[CR22] IAEA (2013). Radiation protection and management of NORM residues in the phosphate industry.

[CR23] IAEA (2018) Naturally occurring radioactive material (NORM VIII). In: Proceedings of an International Symposium.

[CR24] IAEA/NEA (2020) Uranium resources, production and demand. 10.1787/d82388ab-en

[CR25] Imani M, Adelikhah M, Shahrokhi A (2021). Natural radioactivity and radiological risks of common building materials used in Semnan Province dwellings, Iran. Environ Sci Pollut Res.

[CR26] James MM (2019). Impacts of tobacco farming on forest cover in Bukira West/Bukira East location, Migori County.

[CR27] John WA, Lückel B, Matschiavelli N (2022). Endocytosis is a significant contributor to uranium (VI) uptake in tobacco (Nicotiana tabacum) BY-2 cells in phosphate-deficient culture. Sci Total Environ.

[CR28] Kadhim NF, Ridha AA (2019). Radiation hazards of the moassel consumed in Baghdad/Iraq using NaI(Tl) gamma spectroscopy. Int J Environ Sci Technol.

[CR29] Kasoga KF, Mwalongo DA, Sawe SF et al (2015) Ambient gamma dose rate measurements at Manyoni uranium deposits, Singida, Tanzania. Proc SA Inst Phys 180–185

[CR30] Khater AEM (2012). Uranium and trace elements in phosphate fertilizers-Saudi Arabia. Health Phys.

[CR31] Kovacs T, Bator G, Schroeyers W et al (2017) From raw materials to NORM by-products. In: Naturally Occurring Radioactive Materials in Construction. Woodhead Publishing, pp 135–182. 10.1016/B978-0-08-102009-8.00006-2

[CR32] Landsberger S, Lara R, Landsberger SG (2015). Non-destructive determination of uranium, thorium, and 40&nbsp;K in tobacco and their implication on radiation dose levels to the human body. Radiat Prot Dosimetry.

[CR33] Lisuma J, Mbega E, Ndakidemi P (2020). Influence of tobacco plant on macronutrient levels in sandy soils. Agronomy.

[CR34] Lisuma JB, Mbega ER, Ndakidemi PA (2021). The effects of cultivating tobacco and supplying nitrogenous fertilizers on micronutrients extractability in loamy sand and sandy soils. Plants.

[CR35] Lisuma JB, Mbwambo AF (2022) Suitability of Blended N10P18K24 Fertilizer on Flue Cured Tobacco (Nicotiana tabaccum L) Production in Tanzania. Research Report submitted to Tanzania Fertilizer Regulatory Authority (TFRA) on 25 April 2022.

[CR36] Lisuma JB, Muna EI, Mbwambo AF (2022). Suitability of blended Minjingu fertilisers for flue-cured tobacco production in Tanzania’s sandy soils. J Mod Agric Biotechnol.

[CR37] Lisuma JB, Philip AJ, Ndakidemi PA, Mbega ER (2022). Assessing residue effects of tobacco nicotine on the yields, nutrient concentrations and nicotine uptake of a subsequent maize crop. Field Crops Res.

[CR38] Lokuruka MNI (2020). Food and nutrition security in East Africa (Kenya, Uganda and Tanzania): status, challenges and prospects. Food Security in Africa.

[CR39] Lokuruka M (2021). Food and nutrition security in East Africa (Rwanda, Burundi and South Sudan): status, challenges and prospects. Food Security in Africa.

[CR40] Makweba MM, Holm E (1993). The natural radioactivity of the rock phosphates, phosphatic products and their environmental implications. Sci Total Environ.

[CR41] Mkhaiber AF, Al-Bayati AT, Jawad EA, Mahdi KH (2020) Using gamma spectroscopy to calculate radioactive contaminants in foreign and local tobacco samples. J Southwest Jiaotong Univ 55(2):1–7. 10.35741/issn.0258-2724.55.2.31

[CR42] Moberg JR (2001) Soil and plant material analysis manual. The Royal Veterinary and Agricultural University, Chemistry Department, Copenhagen, Denmark Soil and Plant Analysis Manual

[CR43] Mostafa AMA, Uosif MAM, Elsaman R (2020). The dependence of natural radioactivity levels and its radiological hazards on the texture of agricultural soil in Upper Egypt. Environ Earth Sci.

[CR44] Mwalongo DA, Haneklaus NH, Lisuma JB (2022). Uranium in phosphate rocks and mineral fertilizers applied to agricultural soils in East Africa. Environ Sci Pollut Res.

[CR45] Naina M, Gupta M, Chauhan RP (2010). Estimation of radioactivity in tobacco. Indian J Pure Appl Phys.

[CR46] Ndomba HH (2018) A history of peasant tobacco production in Ruvuma Region, Southern Tanzania, c 1930–2016. Dissertation. http://hdl.handle.net/10019.1/103264

[CR47] Newman RT, Lindsay R, Maphoto KP (2008). Determination of soil, sand and ore primordial radionuclide concentrations by full-spectrum analyses of high-purity germanium detector spectra. Appl Radiat Isot.

[CR48] Ngulimi MF, Ishiga H (2016). Geochemical examination of surficial soil overlying uranium deposit in Manyoni, Central Tanzania. Earth Sci Res.

[CR49] Papastefanou C (2009). Radioactivity of tobacco leaves and radiation dose induced from smoking. Int J Environ Res Public Health.

[CR50] Polouckova V (2021) Correction in determination of specific activity of radionuclides by gamma spectrometry in building materials. IOP Conf. Ser.: Mater. Sci. Eng. 1039 012022. 10.1088/1757-899X/1039/1/012022

[CR51] Purnama D, Damayanti T (2020). Determination of internal and external hazard index of natural radioactivity in well water samples. J Phys Conf Ser.

[CR52] ORTEC (2020) GammaVision® Maestro-PRO®. https://www.ortec-online.com/products/application-software/maestro-pro

[CR53] Ratnikov AN, Sviridenko DG, Popova GI, Sanzharova NI, Mikailova RA (2020) The Behaviour of Uranium in Soils and the Mechanisms of Its Accumulation by Agricultural Plants. In: Gupta D, Walther C (eds) Uranium in plants and the environment. Radionuclides and Heavy Metals in the Environment. Springer, Cham. 10.1007/978-3-030-14961-1_5

[CR54] Ribeiro FCA, Silva JIR, Lima ESA (2018). Natural radioactivity in soils of the state of Rio de Janeiro (Brazil): radiological characterization and relationships to geological formation, soil types and soil properties. J Environ Radioact.

[CR55] Shakhashiro A, Trinkl A, Sansone U (2008). The IAEA’s “ALMERA Network” proficiency test on the determination of gamma-emitting radionuclides: a test of results comparability. Appl Radiat Isot.

[CR56] Söğüt Ö, Kocaer AF, Selçuk Zorer Ö (2014). Micro-chemical and radiological characterization using γ-spectrometry and WDXRF spectrometry and annual effective dose of cigarette tobaccos. Microchem J.

[CR57] Sokolik GA, Ovsiannikova SV, Papenia MV (2020) Uranium and its distribution in typical Belarusian soils. In: Uranium in Plants and the Environment. Springer, Cham, pp 33–68

[CR58] Stojanovic MD, Mihajlovic ML, Milojkovic JV (2012). Efficient phytoremediation of uranium mine tailings by tobacco. Environ Chem Lett.

[CR59] Tagami K, Uchida S (2020). Soil-to-crop transfer factor: consideration on excess uranium from phosphate fertilizer. Uranium in Plants and the Environment.

[CR60] Takeda A, Tsukada H, Takaku Y et al (2006) Accumulation of uranium derived from long-term fertilizer applications in a cultivated Andisol. Sci Total Environ 367. 10.1016/j.scitotenv.2006.01.006 10.1016/j.scitotenv.2006.01.006 16487995

[CR61] Taskin H, Karavus M, Ay P (2009). Radionuclide concentrations in soil and lifetime cancer risk due to gamma radioactivity in Kirklareli, Turkey. J Environ Radioact.

[CR62] Tufail M (2012). Radium equivalent activity in the light of UNSCEAR report. Environ Monit Assess.

[CR63] UNSCEAR (2008) Sources and effects of ionizing radiation: United Nations Scientific Committee on the Effects of Atomic Radiation 2008 Report.

[CR64] Van Dung N, Thuan DD, Nhan DD (2022). Radiation exposure in a region with natural high background radiation originated from rare earth element deposits at Bat Xat district, Vietnam. Radiat Environ Biophys.

[CR65] Wanyonyi E, Talibita M, Kirigwajo M et al (2020) Africa Tobacco Industry Monitoring (ATIM). https://pub.nkumbauniversity.ac.ug/xmlui/handle/123456789/910

[CR66] Yamaguchi N, Kawasaki A, Iiyama I (2009). Distribution of uranium in soil components of agricultural fields after long-term application of phosphate fertilizers. Sci Total Environ.

[CR67] Zlobina A, Farkhutdinov I, Carvalho FP (2022). Impact of environmental radiation on the incidence of cancer and birth defects in regions with high natural radioactivity. Public Health.

